# Interpreting character variation in turtles: *Araripemys barretoi* (Pleurodira: Pelomedusoides) from the Araripe Basin, Early Cretaceous of Northeastern Brazil

**DOI:** 10.7717/peerj.9840

**Published:** 2020-09-29

**Authors:** Saulo Limaverde, Rodrigo Vargas Pêgas, Rafael Damasceno, Chiara Villa, Gustavo R. Oliveira, Niels Bonde, Maria E.C. Leal

**Affiliations:** 1 Centro de Ciências, Departamento de Geologia, Universidade Federal do Ceará, Fortaleza, Brazil; 2 Laboratory of Vertebrate Paleontology and Animal Behaviour, Universidade Federal do ABC, São Bernardo do Campo, São Paulo, Brazil; 3 Laboratório de Paleontologia & Sistemática, Área de Ecologia, Departamento de Biologia, Universidade Federal Rural de Pernambuco, Recife, PE, Brazil; 4 Laboratory of Biological Anthropology, Department of Forensic Medicine, Copenhagen University, Copenhagen, Denmark; 5 Section Biosystematics, Zoological Museum (SNM, Copenhagen University), Copenhagen, Denmark; 6 Fur Museum (Museum Saling), Fur, Denmark; 7 Faculty of Health, Aarhus University, Aarhus, Denmark

**Keywords:** Araripe, Sexual dimorphism, Cretaceous, Ontogeny, *Araripemys*, Intraspecific variation, Polymorphism

## Abstract

The Araripe Basin (Northeastern Brazil) has yielded a rich Cretaceous fossil fauna of both vertebrates and invertebrates found mainly in the Crato and Romualdo Formations, of Aptian and Albian ages respectively. Among the vertebrates, the turtles were found to be quite diverse, with several specimens retrieved and five valid species described to this date for the Romualdo Formation. There were also records of turtles from Ipubi and Crato Formations, mainly fragmentary material which precluded proper specific identification; however, *Araripemys barretoi* is supposed to occur on both Crato and Romualdo Formations. Here we describe thirteen specimens of *A. barretoi*-including the first description of an almost complete individual, bearing a skull, from the Crato Formation. We report a great amount of morphological variation, interpreted as being essentially of intraspecific nature, including individual, sexual and ontogenetic variation.

## Introduction

Turtles are remarkable reptiles that possess a bony shell alongside other unique morphological characters, such as a shoulder girdle enclosed by the ribcage. The order Testudines is nowadays split in two monophyletics groups: Pleurodire (side-necked) and Cryptodire (hidden-necked) turtles (Gaffney, 1975; Joyce & Bell, 2004).

Pleurodires are split in two clades: Cheloides and Pelomedusoides (Gaffney, Tong & Meylan, 2006; Sereno & ElShafie, 2013). The Cheloides comprise a single lineage, Pan-Chelidae, ranging from Cretaceous to present day (Lapparent de Broin & De la Fuente, 2001; De la Fuente, 2003; Bona & De la Fuente, 2005; Gaffney, Tong & Meylan, 2006; Maniel & De la Fuente, 2016; De la Fuente et al., 2017), while the Pelomedusoides comprise six families, the extant Pelomedusidae and Podocnemididae, and the extinct Bothremydidae, Euraxemydidae, Peiropemydidae and Araripemydidae (Ferreira et al., 2018).

Araripemydidae was first erected by Price (1973) to accommodate *Araripemys barretoi* from the Romualdo Formation of the Araripe Basin. Later on, Lapparent de Broin (1980) described *Taquetochelys decorata*, from the Elharz Formation, based on shell fragments. This taxon was assigned to the clade Araripemydidae based mainly on shell texture, which was deemed similar to the distinctive pitted ornamentation pattern of *A. barretoi*. The genus was considered as *incertae sedis* within Pelomedusoides for lacking diagnostic features due to the fragmentary state of the material (De la Fuente & Lapparent de Broin, 1997; Gaffney, Tong & Meylan, 2006) and was regarded as a *nomen dubium* by Sereno & ElShafie (2013). Fielding, Martill & Naish (2005) described a new species for the genus *Araripemys* (*A*. ‘*arturi’*) based on fragmentary material from the Crato Formation (Araripe Basin), but the taxon was deemed poorly established and ended synonymised with *A. barretoi* in subsequent reviews (Gaffney, Tong & Meylan, 2006; Oliveira & Kellner, 2007). Araripemydidae was thus a redundant taxon until the description of *Laganemys tenerensis* Sereno & ElShafie (2013), based on an almost complete skeleton from the Elharz Formation of Niger, which was allocated within this family. Recently, Pérez-García (2018) has proposed a synonymisation between *T. decorata* and *L. tenerensis*, retaining the species within Araripemydidae.

The genus *Araripemys* is represented by the single species *A. barretoi*. The holotype and most of the specimens known come from the Romualdo Formation (Early Albian) of the Araripe basin (Price, 1973; Kischlat & de Almeida Campos, 1990; Schleich, 1990; Meylan, 1996; Gaffney, Tong & Meylan, 2006), but there are records also for the Crato Formation (Late Aptian) of the same basin (e.g. Fielding, Martill & Naish, 2005; Oliveira & Kellner, 2005, 2017; Romano et al., 2013) and for the Itapecuru Formation (Albian) of the Parnaiba Basin (Batista & Carvalho, 2007). There is a large number of specimens retrieved from the Romualdo Formation nodules displaying the characteristic 3D preservation of this formation, with several of them exhibiting both cranial and post-cranial elements. However, for the Crato Formation the only known specimens are, so far, a fragment of a shell along with a hindlimb which was the holotype of *A*. ‘*arturi’* (Fielding, Martill & Naish, 2005); an isolated plastron (Oliveira & Kellner, 2005); two partially complete juvenile specimens displaying incomplete skulls assigned to *Araripemys* cf. *A. barretoi* (Oliveira & Kellner, 2017); and an almost complete post-cranial skeleton (SMNK, no collection number provided) which was figured by Naish (2007), but remains undescribed. No complete specimen has ever been described for the Crato Formation so far.

Morphological variation remains the keystone over which most of the natural sciences are built. Following the very didactic paper by Grande (2004), there are three main categories of morphological variation: taxonomic, ontogenetic and individual.

Pritchard (1988) has already drawn attention to how uneven are the studies on intraspecific variation in turtles, with a ‘frustratingly incomplete’ literature concerning extant species. Adding to the problem, palaeontologists usually have to study shell-only material, as it is the most common form of preservation: complete specimens retaining the skull are a rare find. Using *A. barretoi* as an example, for which dozens of shell-only specimens are known (the holotype, for instance, is a shell-only material), there are only four nearly complete skulls described (Gaffney, Tong & Meylan, 2006).

The identification of morphological variations in fossil turtles has important implications for phylogenetic analysis. The diagnostic features presented by Fielding, Martill & Naish (2005) to erect a new *Araripemys* species, *A. ‘arturi’*, were invalidated, those being: (1) ovoid-shaped carapace, whereas *A. barretoi* would display a posterolateral angulation at the carapace lateral margin (Price, 1973; Meylan, 1996); (2) peripherals 9 and 10 equally long as wide; and (3) lack of arrow-shaped pedal unguals, supposedly characteristic of *A. barretoi* after Meylan (1996). The first has been proposed to be taphonomical or dubious due to the material incompleteness; while the second could represent ontogenetic, sexual or individual variation; and the third, in turn, has been proposed to represent individual variation (Gaffney, Tong & Meylan, 2006; Oliveira & Kellner, 2007; Pérez-García, 2018). Although the new species were synonymised with *A. barretoi*, the very nature of the features claimed were never elucidated. The knowledge of morphological variation due to sexual dimorphism as well as individual and ontogenetic variation will allow a refinement of the characters used for phylogenetic or systematic purposes, at least at the species taxonomic level.

The turtle shell presents a limited number of characters and shows a wide spectrum of individual variation, the most usual variation occurring on the neural bones of the carapace (Romano et al., 2013). The supposed ancestral condition of eight predominantly hexagonal neurals can be modified either by proliferation or reduction of elements in the series; however, it was suggested that neomorphic elements occur rarely, while elimination and subsequent reduction in neural numbers would be far more frequent (Pritchard, 1988). Changes occur usually at the end of the series, by loss of exposed neurals, by obliteration (when the neurals are present but covered by the surrounding bones), and by changes in shape (Pritchard, 2008). Intraspecific variation of neural bones is widespread among living taxa, but very unevenly documented (Pritchard, 1988, 2008, and references therein). It was first reported for *A. barretoi* by Meylan (1996) concerning the contacts of the last neural. The plastron shows less variation in its number of components than the carapace, but displays as much or even more variation in shape (Pritchard, 2008).

Another important source of intraspecific variation, this one well established in living forms but sometimes not so easy to determine in the fossil record, is sexual dimorphism. It can be observed in turtles usually relative to shell and plastron features. On sexual dimorphism in recent and fossil taxa see for example Lagarde et al. (2001), Lapparent de Broin, De la Fuente & Fernandez (2007), Pritchard (2008), Cadena, Jaramillo & Bloch (2013), and Cadena (2015), among others.

The goal of this work is to describe 14 new specimens of *A. barretoi* (three from the Crato Formation and 11 from the Romualdo Formation) and reexamine other five (one from the Crato Formation and four from the Romualdo Formation), and to report all morphological variation, establishing patterns of intra-specific variation for this species. Such approach has never been done before for *A. barretoi*, despite the high number of known specimens. This new data will add to the current knowledge on the morphology of *A. barretoi* and will allow a refinement of the characters used in systematic analyses.

## Geological Setting

The Araripe Basin is a sedimentary basin whose origin is related to the breakage of Gondwana and the opening of the South Atlantic Ocean during the Early Cretaceous. This intracratonic basin is located on the borders of the states of Ceará, Pernambuco and Piauí in Northeastern Brazil. It has an area of approximately 9,000 km^2^ encompassing not only the Araripe Plateau but also the Cariri Valley (Valença, Neumann & Mabessone, 2003).

The Santana Group is the most fossiliferous unit, and includes the Crato and Romualdo Formations. These are two *konservat lagerstätten* famous for their taxonomic diversity and extraordinary preservation of organic structures, including remarkable soft tissues (e.g. Martill, 1988; Martill, Bechly & Loveridge, 2007; Campos & Kellner, 1997; Kellner, 1996a, 1996b; Pinheiro et al., 2012; Maldanis et al., 2016). However, there is not much agreement concerning its stratigraphy or age, which is usually estimated as Aptian–Albian; the reader is referred to Martill (2007) for a review. In this work we use the stratigraphic framework of Valença, Neumann & Mabessone (2003), with the Santana Group being constituted, from the bottom to the top, by the Rio Batateira, Crato, Ipubi, Romualdo and Arajara Formations.

## Materials and Methods

### Material

#### Newly described material

LP-UFC 722; MPSC R 010 (part and counterpart); MPSC R 2107; UFRPE 5302; MN 6949-V*; MN 6743-V; MN 6744-V; DGM 346-LE; DGM 1449-R; MPSC R 2308; MPSC R 874; MPSC R 134 (part and counterpart); MPSC R 137; SMNK PAL no number.

#### Revisited material

DGM 765-R (holotype of *A. barretoi*); MN 6637-V (first described by Kischlat & de Almeida Campos, 1990); SMNK PAL 3979 (holotype of *A. arturi*); BSP 1977 I 1 (first reported by Schleich (1990)); BSP 1981 I 38.

#### Comparative material

*Chelonia mydas:* UFRPE 5382; 5383 and 5384; *Pelomedusa galeata*: KU SNM-CN 195; *Pelomedusa subrufa*: KU SNM-CNR2821; *Pelusios derbianus*: KU SNM-CN192; *Hydromedusa maximiliani*: KU SNM-CN211; *Phrynops hilarii*: KU SNM-CN 214; KU SNM-CN 266; *A. barretoi*: MPSC R 135; MPSC R 136; MPSC R 778; MPSC R 873; MPSC R 1305; MPSC R 2309; MN 6745-V; MN 4893-V; MN 7191-V; AMNH 24452; AMNH 24453; AMNH 24454; AMNH 24456; AMNH 30651; THUg 1357; THUg 1907.

From the literature: MB.R.3448; SMNK no number (Naish, 2007).

All specimens used in this study are housed in public scientific collections.

*The specimen MN 6949-V was unfortunately lost during the fire that destroyed the Brazilian National Museum in the night of September 2nd 2018. The illustrations we present here are all that was left from it.

### Preparation

Mechanical and chemical preparation were conducted by us only in the following specimens: LP-UFC 722; MPSC R 010 (counterpart); MPSC R 2107 and UFRPE 5302. The protocol modified from Toombs & Rixon (1959) was adapted to particularities of each specimen following Leal & Brito (2004) and Silva & Kellner (2006). After chemical preparation, the specimen LP-UFC 722 underwent also a consolidating protocol following Cnudde et al. (2007).

### Image acquisition, processing and analysis

The specimen LP-UFC 722 was scanned using a Siemens Somatom Definition CT scanner, at the Department of Forensic Medicine, University of Copenhagen. After chemical/mechanical preparation, the entire specimen was scanned with the following settings: 140 kV, 550 mAs, 0.6 mm slice thinness, 0.4 pitch, 0.3 slice increment and a sharp reconstruction algorithm (H70h). A field of view of 182 mm was used resulting in images with a pixel size of 0.35 mm.

A detailed scanning of the skull was also performed using the same settings, but reducing the field of view to 93 mm. The resulted pixel size of the CT images was 0.18 mm. The CT scanning data processed using the image software Mimics (Materialise) version 12. A 3D model of the skull was created by using data segmentation methods described by Lynnerup (2007) and Villa & Lynnerup (2012).

## Results

**Systematic Palaeontology**

Testudines Batsch, 1788

Pleurodira Cope, 1864

Pelomedusoides Cope, 1868

Araripemydidae Price, 1973

*Araripemys* Price, 1973 *Araripemys barretoi* Price, 1973

**Synonymy**. *Araripemys arturi* Fielding, Martill & Naish, 2005

**Holotype**. DGM 756-R, incomplete shell (cast MN 6945-V).

**Locality and Horizon**. Outcrops of the Crato (Aptian–Albian) and Romualdo Formations (Albian), in the Santana Group of Araripe Basin, Northeastern Brazil.

**Revised diagnosis**. Pelomedusoid with extensive temporal and cheek emargination; basisphenoid very long nearly reaching palatines; narrow triturating surface without accessory ridges; thin labial ridge; incisura columela auris not enclosed by bone; fossa precolumellaris deep; quadrate-basioccipital contact absent; ventral exposure of the occipital extensive; prootic widely exposed ventrally; processus interfenestralis of opisthotic widely exposed ventrally; processus paraoccipitalis of the opisthotic surpassing by far the supraoccipital crest; very long neck; postatlantal cervical vertebrae with completely fused postzygapophyses; first thoracic strongly sutured to the nuchal; medial and lateral centralia absent; flattened shell; fine ornamentation consisting variably of pits and/or ridge-and-sulcus configurations; first costals separating nuchal from first peripherals; small and subtriangular peripheral 1; neural series including 8–10 neurals; persistence of costal fontanelles in mature forms; reduced cruciform plastron with well-developed axillary and inguinal buttresses; inverted V-shaped entoplastron; J-shaped epiplastra forming a sharp point anteriorly; absent mesoplastra; absent gular scutes; three midplastral fontanelles.

**Description**

The full, detailed and individual descriptions of all newly reported and redescribed specimens can be found in the Supplemental Material. In this section we combine and compile data on all specimens (mentioning them wherever appropriate) focusing on new morphological data and on observed variations. These data comprise features of the skull, shell, and unguals. Variations are also compiled in Table S1 of Supplemental Material 2.

**Figure 1:**
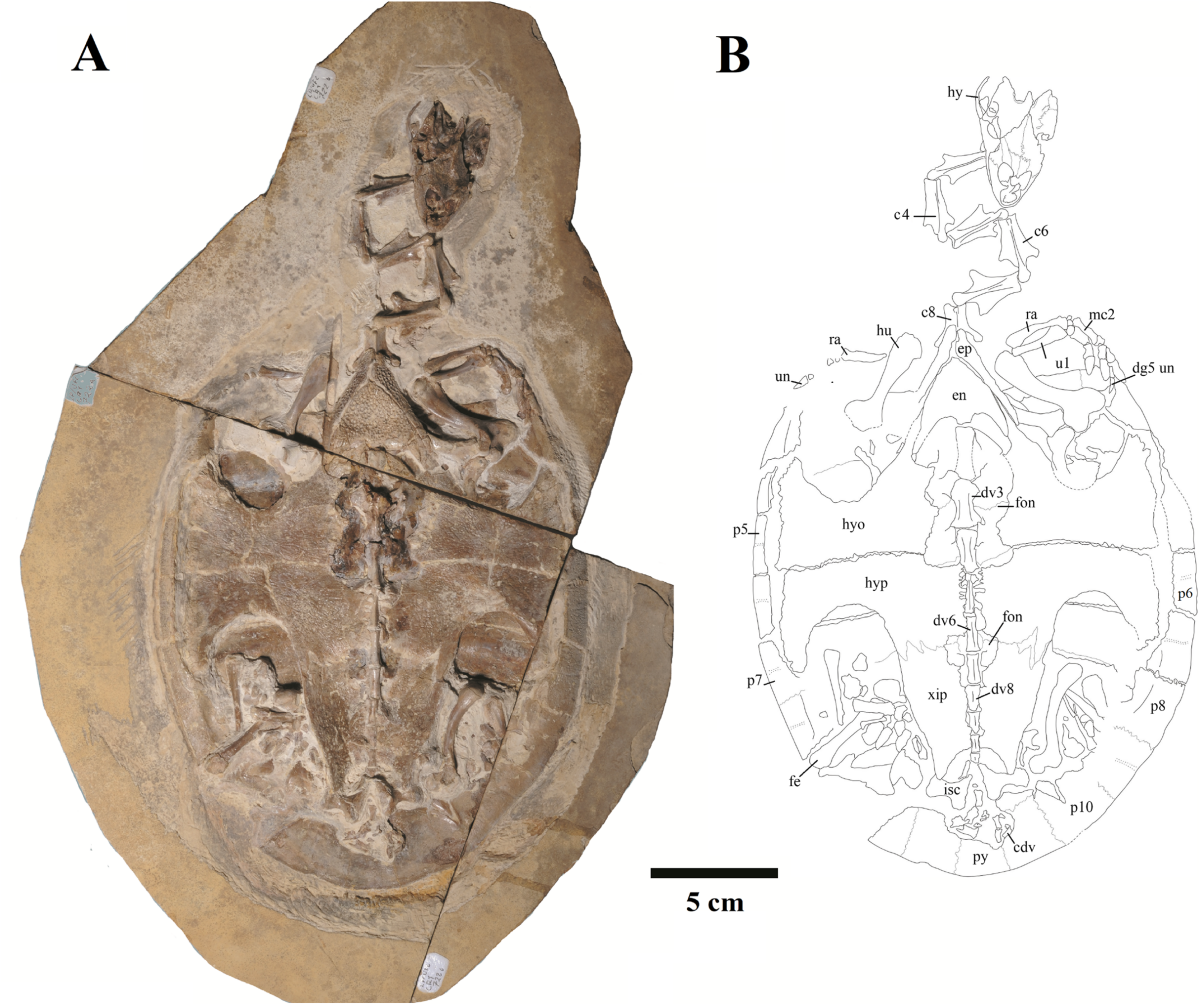
Specimen LP-UFC 722; whole specimen. (A) Photograph; (B) schematic drawing. Photo by Marcus Krag. Drawing by Thales Nascimento. Abbreviations: c, cervical vertebrae; cdv, caudal vertebrae; dg, digit; dv, dorsal vertebrae; en, entoplastron; ep, epiplastron; fe, femur; fon, fontanelle; hu, humerus; hy, hyoid; hyo, hyoplastron; hyp, hypoplastron; isc, ischium; mc, metacarpal; p, peripherals; py, pygal; ra, radius; ul, ulna; un, ungual; xip, xiphiplastron.

**Figure 2:**
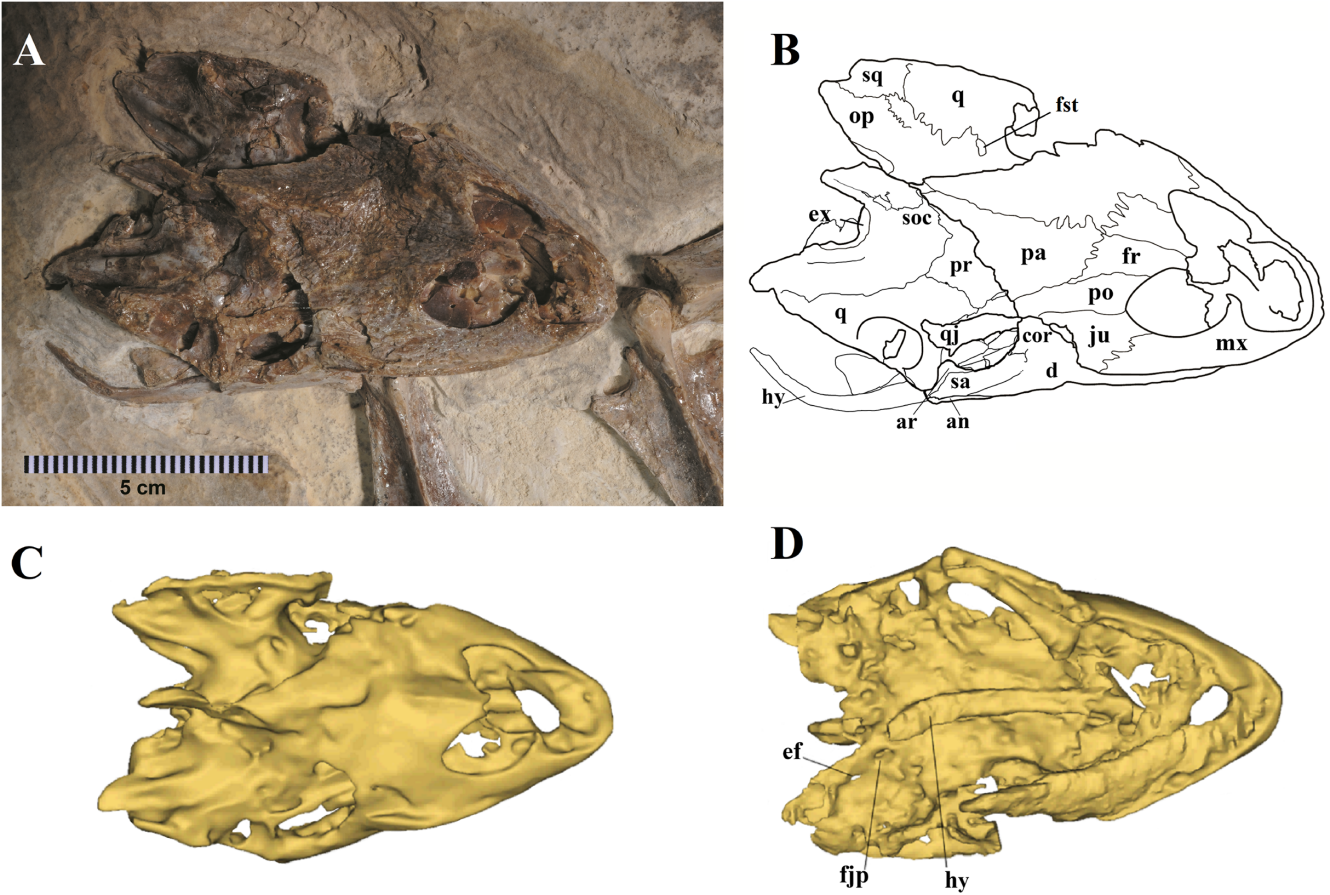
Specimen LP-UFC 722; skull. (A) Skull in dorsal view; (B) schematic drawing; (C) 3D-model in dorsal view; (D) 3D-model in ventral view. Photo by Marcus Krag. Drawing by Thales Nascimento. Abbreviations: an, angular; ar, articular; cor, coronoid; d, dentary; ef, extra foramen; ex, exoccipital; fjp, foramen jugulare posterius; fr, frontal; fst, foramen stapedio temporale; hy, hyoid; ju, jugal; mx, maxillary; op, opistotic; pa, parietal; po, postorbital; pr, prootic; q, quadrate; qj, quadratojugal; sa, surangular; soc, supraoccipital; sq, squamosal.

**Figure 3:**
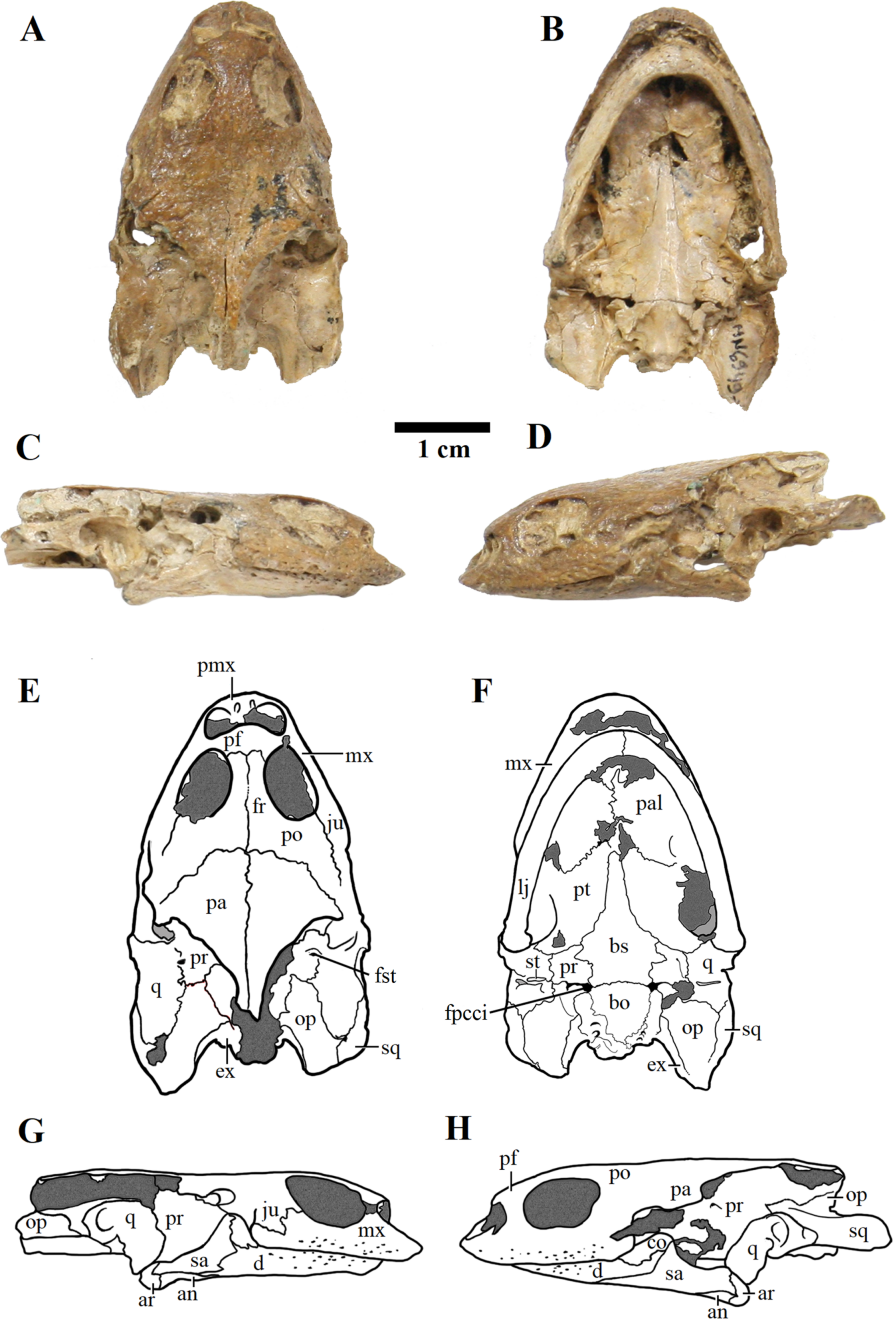
MN 6949-V. Skull in (A) dorsal; (B) ventral; (C) right; and (D) left views. (E–H), respective schematic drawings. Photos by Rodrigo V. Pêgas and Thales Nascimento. Drawings by Maria E.C. Leal and Rodrigo V. Pêgas, based on drawing by Niels Bonde. Abbreviations: an, angular; ar, articular; bo, basioccipital; bs, basisphenoid; cor, coronoid; d, dentary; ex, exoccipital; fpcci, foramen posterius canalis carotici interni; fr, frontal; fst, foramen stapedio temporale; ju, jugal; lj, lower jaw; mx, maxillary; op, opisthotic; pa, parietal; pal, palatine; pf, prefrontal; pmx, premaxilla; po, postorbital; pr, prootic; pt, pterygoid; q, quadrate; sa, surangular; sq, squamosal; st, stapes.

**Figure 4:**
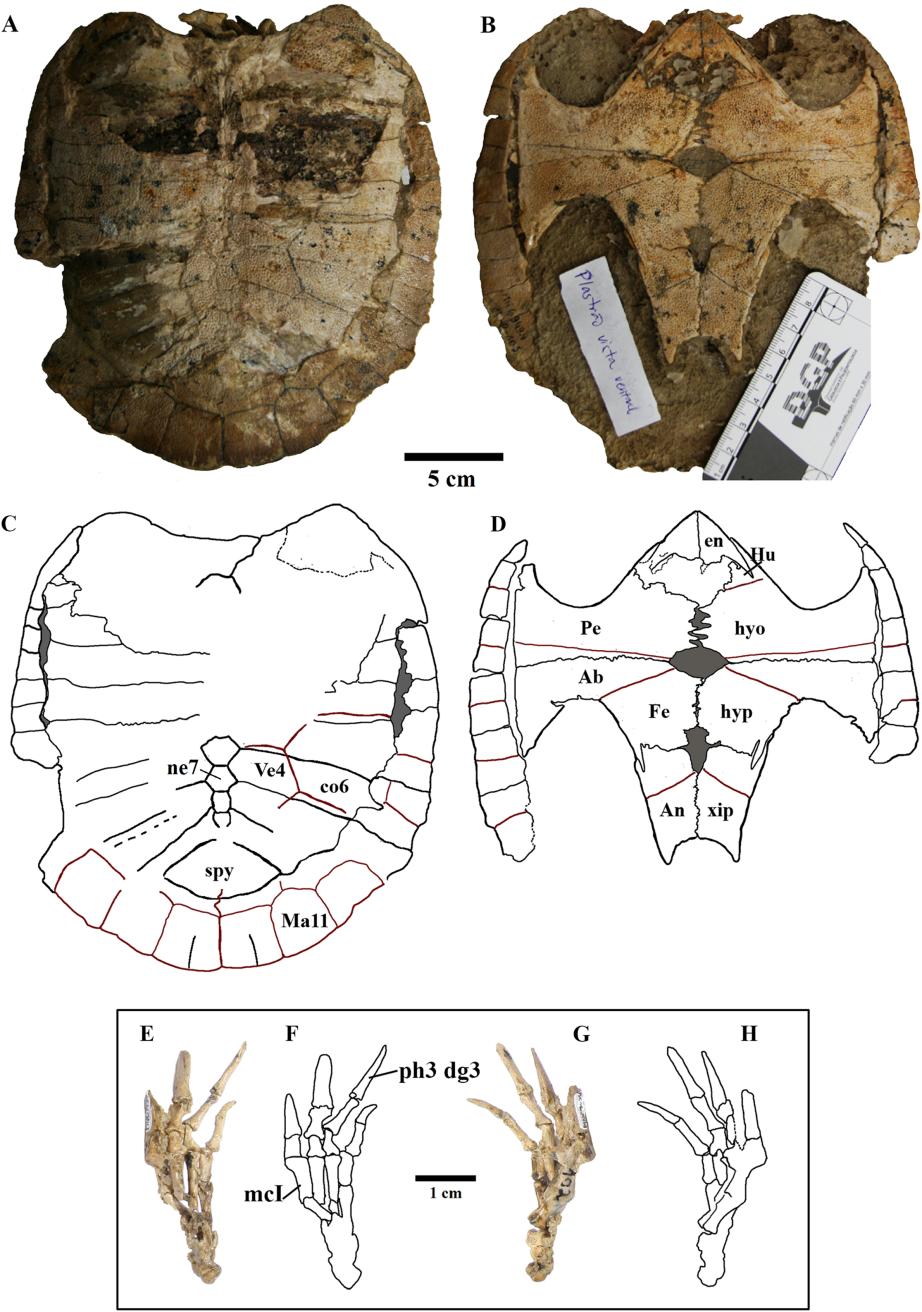
MN 6949-V. Postcranium. (A) Carapace; (B) plastron; (C and D), respective schematic drawings; (E) right manus in dorsal view and (F) schematic drawing; (G) ventral view; with (H) schematic drawing. Photos by Rodrigo V. Pêgas and Thales Nascimento. Drawings by Maria E.C. Leal and Rodrigo V. Pêgas. Abbreviations: Ab, abdominal scute; An, anal scute; co, costal; dg, digit; en, entoplastron; Fe, femoral scute; Hu, humeral scute; hyo, hyoplastron; hyp, hypoplastron; Ma, marginal; mc, metacarpal; n, neural; Pe, pectoral scute; ph, phalanx; spy, suprapygal; Ve, vertebral scute; xip, xhiphiplastron.

We present here data on 14 new specimens, as well as revisit five previously reported specimens. The new specimens are, from the Crato Formation: LP-UFC 722 (almost complete specimen exposed in ventral view, with a skull in dorsal view; Figs. 1 and 2), MPSC R 2107 (almost complete plastron in ventral view; Fig. S1), UFRPE 5302 (posterior half of carapace in dorsal view; Fig. S1); and from the Romualdo Formation: MN 6949-V (almost complete shell and skull, plus hyoids, two cervical vertebrae, and a complete manus; Figs. 3 and 4), MPSC R 2308 (entire shell; Fig. S2), DGM 346-LE (almost complete shell; Fig. S2), MN 6744-V (almost complete shell, two cervical vertebrae; Fig. S2), MN 6743-V (partial shell; Fig. S3), DGM 1449-R (carapace; Fig. S4), MPSC R 010 (entire shell), MPSC R 874 (almost complete carapace), MPSC R 134 (carapace; Fig. S4), MPSC R 137 (carapace; Fig. S4), SMNK PAL no number (carapace, cervicals and skull; Fig. S4). We further revisit specimens DGM 756-R (partial shell and femora, holotype; Fig. S2), MN 6637-V (carapace impression, plastron, several post-cranial elements including an ungual; Fig. S3), SMNK PAL 3979 (partial shell and hindlimb, holotype of *A. “arturi”*; Fig. S3), BSP 1977 I 1 (almost complete shell with a few postcranial elements, including an undescribed ungual; Fig. S3) and BSP 1981 I 38 (almost complete shell; Fig. S3).

**Skull**

The skull of *A. barretoi* has already been described in detail by Meylan (1996) and Gaffney, Tong & Meylan (2006). We present here two new well-preserved skulls, providing further data on its morphology and character variation.

The skull of LP-UFC 722, from the Crato Formation, is almost complete and exposed in dorsal view, but some details from the ventral view can be assessed from the CT scan-generated 3-D model (Fig. 2). Overall, the pattern of bone elements and contacts is similar to what has already been described for *A. barretoi*. This skull exhibits an ‘extra foramen’ in the opisthotic–exoccipital suture (Fig. 2D), similar to AMNH 24454 (Gaffney, Tong & Meylan, 2006) and unlike the other known skulls. The foramen jugulare posterius is clearly open as seen in the CT scan-generated 3-D model (Fig. 2D). Both hyoids are preserved, the right one being exposed and the left one hidden ventral to the skull. It can be seen in the CT scan-generated 3-D model. The files for the slices of the CT scan can be assessed through a link available in the Supplemental Material 1.

MN 6949-V includes a three-dimensional, well-preserved skull from the Romualdo Formation (Fig. 3). Its general morphology is also consistent with that previously described (Meylan, 1996; Gaffney, Tong & Meylan, 2006). Differently from LP-UFC 722, its foramen jugulare posterius is completely enclosed by a bony bridge and the ‘extra foramen’ is absent. This specimen further exhibits the stapes preserved in natural position (Fig. 3), conforming the previous identification of the incisura collumela auris by Gaffney, Tong & Meylan (2006). A case of asymmetry can be seen regarding this bone. In MN 6949-V, the right coronoid is quite small and forms the posterior half of the coronoid process. The anterior half is formed almost entirely by the dentary, with only a slender dorsal projection of the coronoid, similarly to what is seen in AMNH 24454 and THUg 1907 (Gaffney, Tong & Meylan, 2006). The left coronoid, on the other hand, extends onto and occupies entirely the anterior half of the process. Again, the same morphology is seen in AMNH 24454 and in THUg 1907 the coronoid occupies most of the anterior half of the coronoid process (Gaffney, Tong & Meylan, 2006).

These two skulls are the most complete up to now, especially MN 6949-V. This specimen shows quite clearly the presence of a considerable premaxillary prognathism in *A. barretoi* (Fig. 3). They both further reveal the presence of an extensive foraminization in the surfaces of the premaxillae, maxillae and dentaries, presumably neurovascular and related to the horny beak.

**Carapace**

**Figure 5:**
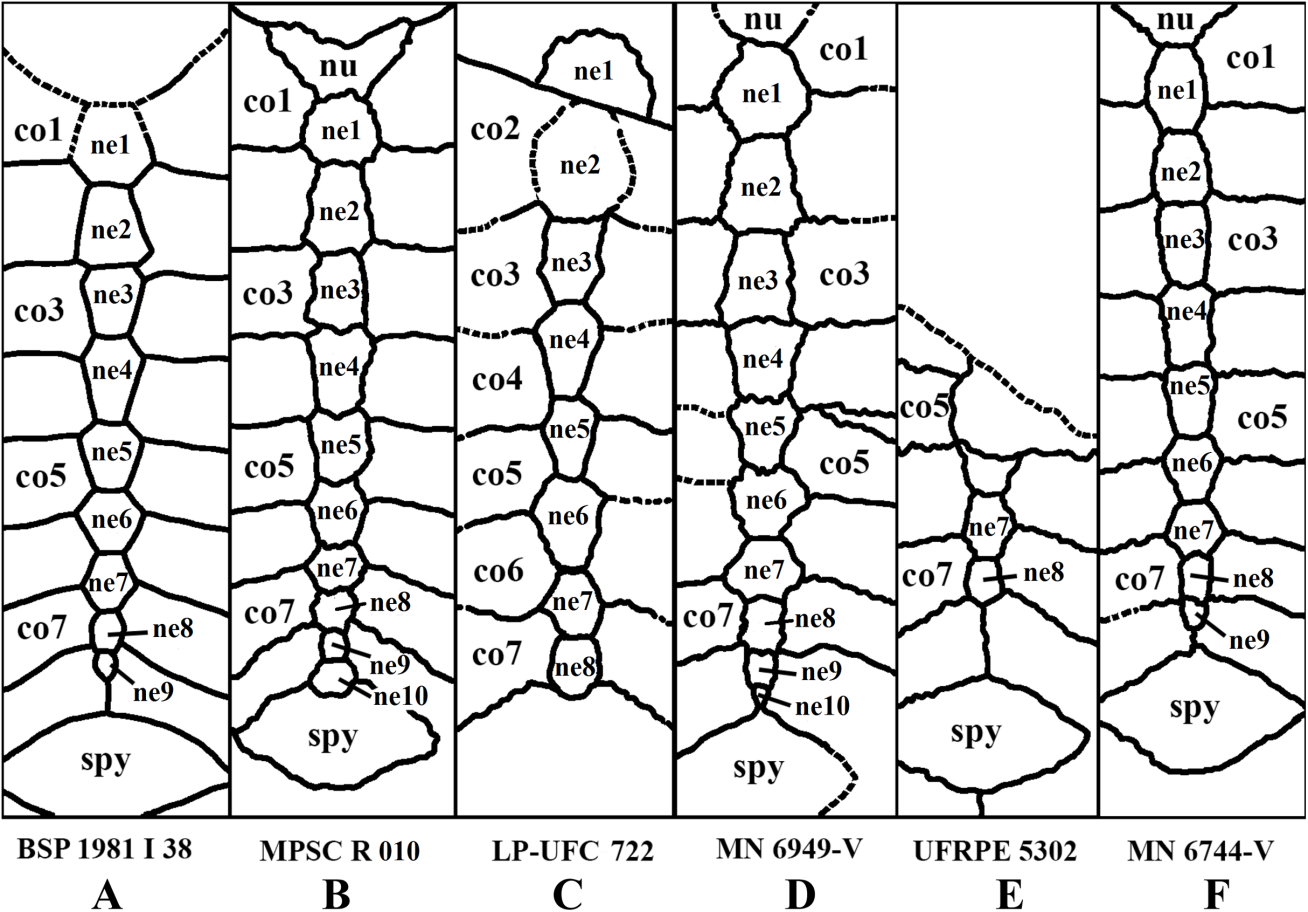
Comparative drawing of neural series from different specimens. (A) BSP 1981 I 38; (B) MPSC R 010; (C) LP-UFC 722; (D) MN 6949-V; (E) UFRPE 5302; (F) MN 6744-V. Drawings by Maria Eduarda C. Leal. Abbreviations: co, costal; ne, neural; nu, nuchal; spy, suprapygal.

**Elements and contacts. **The composition of the carapace in *A. barretoi* varied in the observed specimens mainly in respect of the number of neurals, varying from eight to 10 (see Table S1; Fig. 5). Aside from that, all observed specimens exhibit a nuchal, eight pairs of costals, 11 pairs of peripherals, a pygal and a suprapygal. In all specimens where the region could be observed, the nuchal was separated from the first pair of peripherals by the first pair of costals. Concerning also the contacts between the neurals and surrounding elements, the neural series shows great variation (Table S1). In specimens MN 6744-V, UFRPE 5302 and probably DGM 1449-R there was no contact between the last neural and the suprapygal, with the last pair of costals slightly meeting medially. Finally, the pygal also showed some variation. In specimen UFRPE 5302 the pygal has a unique triangular shape and there is no contact between the pygal and suprapygal, with the last pair of peripherals meeting dorsally to the pygal.

**Costal fontanelles. **The specimens showed variation concerning the relative sizes of the costal fontanelles. The smallest of all specimens herein described (MPSC R 137) shows quite large fontanelles (Fig. S4E). Larger individuals tend to exhibit relatively larger costals and smaller fontanelles. The two largest specimens (DGM 1449-R; MPSC R 134) only show diminutive fontanelles (Fig. S4A–S4C).

**Shape. **The shape of the carapace in *A. barretoi* is known to vary from the squared morphology described by Price (1973) and Meylan (1996) to the oval morphology seen in SMNK PAL 3979 (Fielding, Martill & Naish, 2005). The new specimens herein presented exhibit a variety of shapes, including the oval shape and distinct forms of squared carapaces (Fig. 4; Fig. S2). Oval-shaped carapaces can be seen in specimens LP-UFC 722, DGM 346-LE, MN 6744-V and MPSC R 134, while specimens MN 6949-V, DGM 1449-R, MPSC R 010, MPSC R 874, MPSC R 135 and UFRPE 5302 exhibit all squared-carapaces. In the holotype, the marked angulation that gives the carapace a squared outline is formed between peripherals 8 and 9, as in specimens MN 6949-V and MPSC R 134. In specimen DGM 1449-R, this angulation is formed between peripherals 9 and 10, and in UFRPE 5302, MPSC R 137 and MPSC R 010, between peripherals 7 and 8.

**Ornamentation. **The ornamentation of nuchals, neurals and costals surface did not vary between the observed specimens. In the carapace, only peripherals exhibited variation in, with specimens DGM 1449-R and LP-UFC 722 exhibiting a combination of pits and ridge-and-sulcus pattern in such elements.

**Plastron**

**Figure 6:**
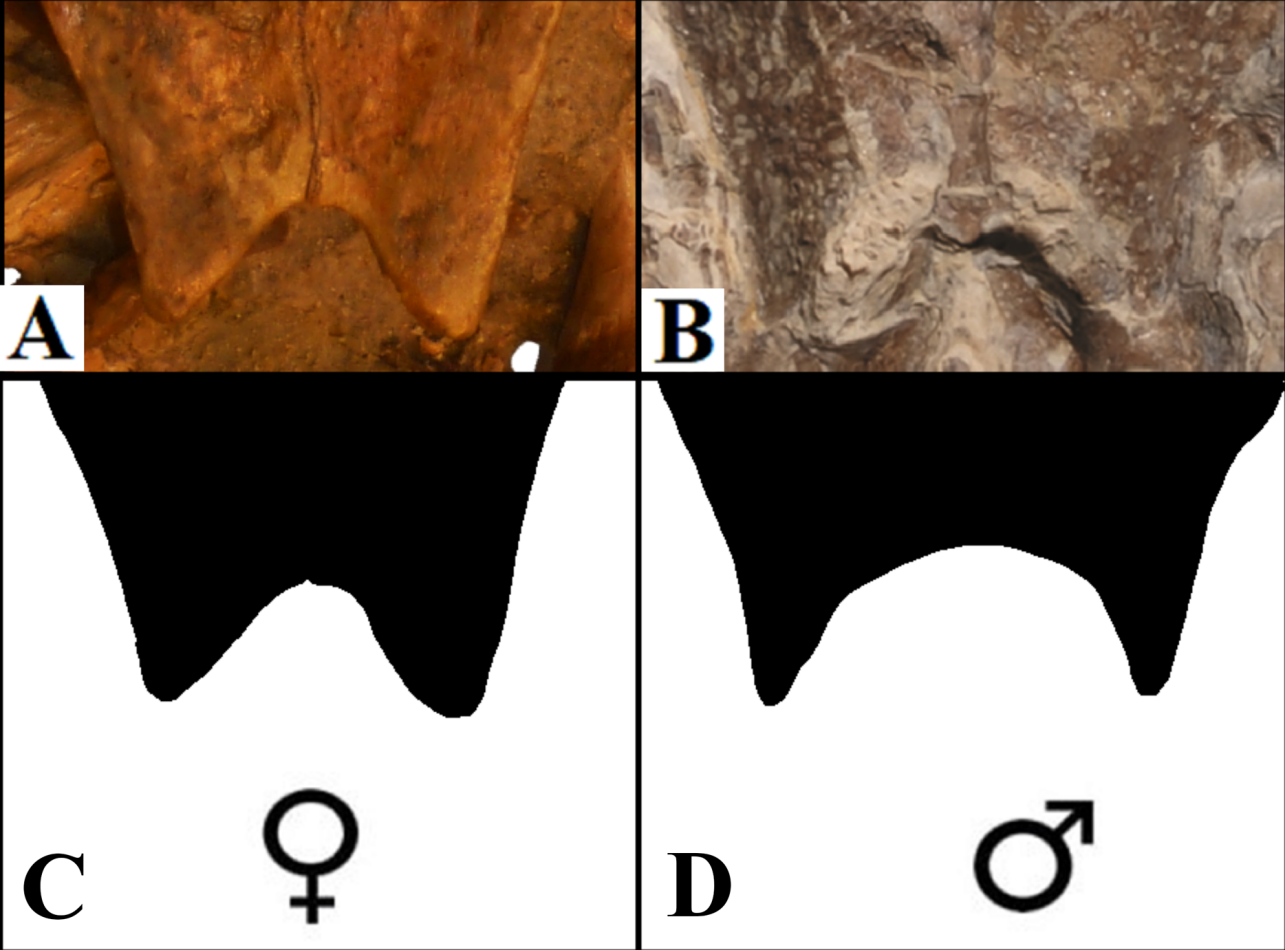
Anal notch variation. (A) V-shaped morphology of the inferred females, as seen in BSP 1981 I 38; (B) U-shaped morphology of the inferred males, as seen in LP-UFC 722; (C and D) schematic drawings by Saulo Limaverde and Rodrigo V. Pêgas.

**Anal notch. **Schleich (1990) described two specimens attributed to *A. barretoi* which display distinct anal notch morphologies (Fig. 6), one which is U-shaped (BSP 1977 I 1) and another V-shaped (BSP 1981 I 38). The V-shaped anal notch can be further seen in specimens MPSC R 010 and MPSC R 874, while the U-shape can be seen in LP-UFC 722, MPSC R 2107, MPSC R 2308, MN 6949-V, DGM 756-R and MN 6637-V.

**Figure 7:**
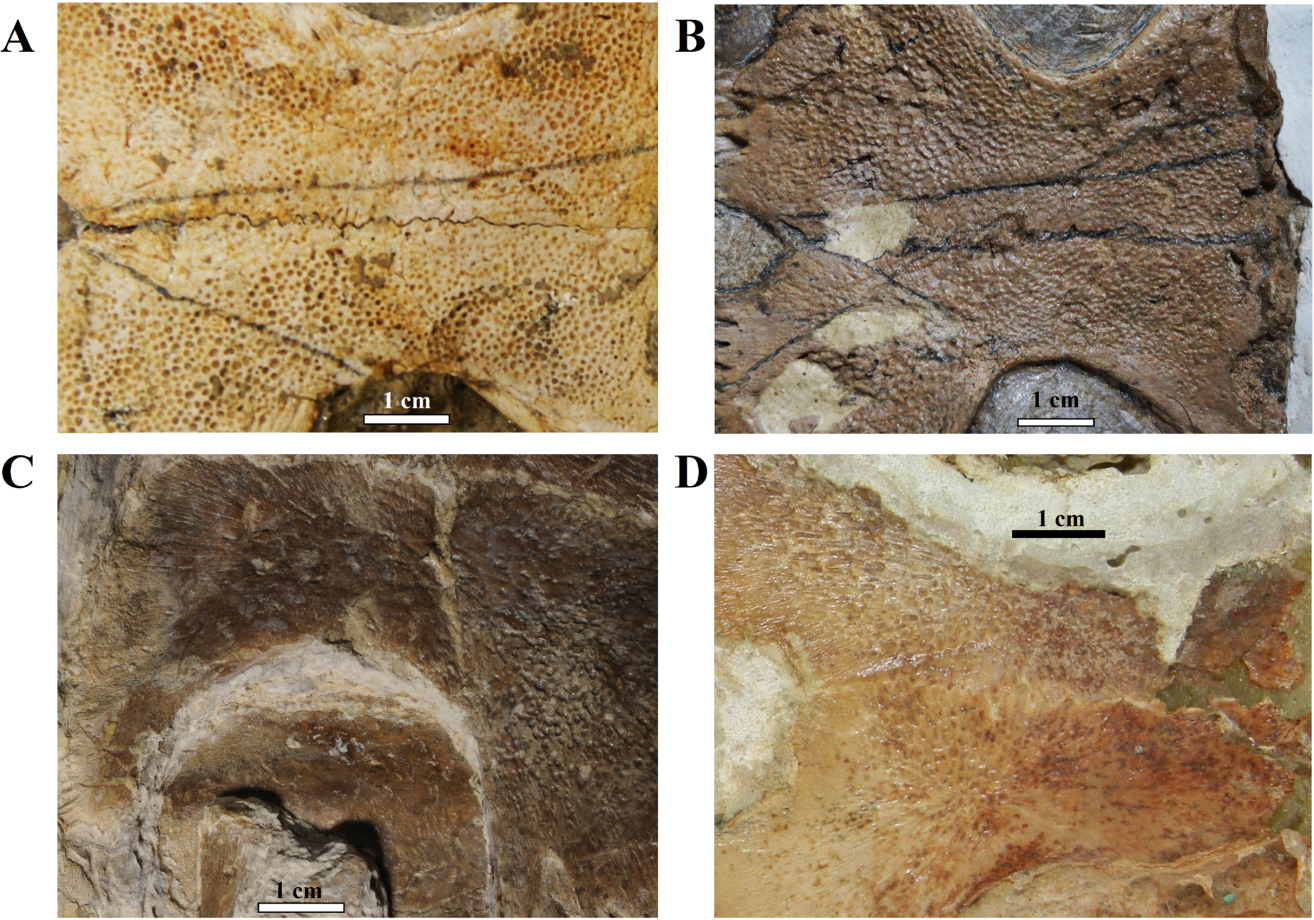
Ornamentation variation in the ventral surface of the hyo- and hypoplastra. Pitted pattern, as seen in (A) MN 6949-V, left side and (B) DGM 346-LE, left side. Combined pitted + ridge-and-sulcus patterns, as seen in (C) LP-UFC 722, right side and (D) MN 6637-V, left side.

**Ornamentation. **All observed epi- and entoplastra exhibited a pitted ornamentation. On the other hand, there was variation in the pattern seen in hyo-, hypo- and xiphiplastra (Fig. 7). Specimens DGM 756-R, SMNK-PAL 3979, MN 6949-V, DGM 364-LE, MPSC R 010 and MPSC R 2308 exhibited a pitted ornamentation in these elements, while the same elements showed a combination of pits and ridge-and-sulcus ornamentation in specimens LP-UFC 722 and MN 6743-V. Specimen MPSC R 2107 exhibited exclusively ridge-and-sulcus ornamentation in these elements, with no signs of pits.

**Unguals. **Variation in the unguals of specimens attributed to *A. barretoi* has already been reported, varying from arrow-head shaped in AMNH numbers 24453, 24454 and 24456 (Meylan, 1996) to simple unguals in SMNK PAL 3979 (Fielding, Martill & Naish, 2005) and MN 6949-V (Oliveira & Kellner, 2007). Here, we report on the unguals of MN 6637-V and LP-UFC 722 (see Supplemental Material), which are arrow-head shaped, and of MPSC R 010 and BSP 1977 I 1 (see Supplemental Material), which are simple.

## Discussion

We have presented above a plethora of morphological variation within specimens of *A. barretoi*. Some of these variations are easily recognisable as intraspecific (e.g. ontogenetic changes, or individual variations, like polymorphisms and sexual dimorphism), while others pose challenges over their interpretations. Accurate discrimination of intra- and interspecific variations in the fossil record can be difficult, especially when control provided by extant analogues is inexistent or insufficient.

Understanding of variation seen in extant species should serve as a control for interpreting variation in the fossil record. Sometimes, osteological variation in extant turtles can be extensive, as what is seen in the orbital margin of *Chelonia mydas* (Cryptodira: Cheloniidae). Two specimens of *C. mydas*, a female and a male (respectively UFRPE 5383 and 5384) are here illustrated, and topographic variation can be observed on some of the skull bones (Fig. S5). On the female skull, the frontal composes a significant part of the orbital margin, standing between the pre-frontal and the post-orbital. However, on the skull of the male specimen, the frontal does not take part on the orbital margin; instead, the contact between the pre-frontal and post-orbital precludes the frontal from reaching the orbit.

In this section, we discuss the diversity and plausible explanation for all variations reported. One must bear in mind that not always the condition found/described in a holotype or figured specimen turns out to be the typical condition observed in a larger sample; see, for instance, the case of *Amia calva* and the single vs paired parietals (Grande, 2004).

### Sexual dimorphism: anal notch

It has been addressed several times in the literature the common sexual variation in turtles concerning the anal notch width measurements (Willemsen & Hailey, 2003; Kaddour et al., 2008; Djordjevic, 2014; Ceballos & Iverson, 2014). Translating such morphometric character into morphological terms, one can say that under this variation, female anal notches can be recognised as V-shaped, while those of males are U-shaped, with a slender terminus of the xiphiplastra.

Sexual dimorphism expressed in the xiphiplastra was already reported for *A. barretoi* (Schleich, 1990) and other fossil pleurodires, for instance, on the platychelyids *Notoemys zapatocaensis*, *Notoemys laticentralis* and *Platychelys oberndorferi* (Pritchard, 2008; Cadena, Jaramillo & Bloch, 2013; Cadena, 2015; Sullivan & Joyce, 2017).

The specimens of *A. barretoi* BSP 1981 I 38, MPSC R 010 share the V-shaped anal notch (Fig. 6) indicative of females. By contrast, LP-UFC 722, MPSC R 2107, MPSC R 2308, MPSC R 874, DGM 756-R, MN 6637-V, MN 6949-V and BSP 1977 I 1 have xiphiplastra with U-shaped anal notch (Fig. 6) indicating that these specimens represent males for the species.

### Individual variation: polymorphisms

#### Skull

As previously reported by Gaffney, Tong & Meylan (2006), there exists in the specimens referred to *A. barretoi* variation in the condition of the foramen jugulare posterius, which varies from completely open to completely closed (Gaffney, Tong & Meylan, 2006). The following conditions are known: completely open for the juvenile specimen THUg 1357 and the subadult specimens AMNH 24453, AMNH 24454 (Gaffney, Tong & Meylan, 2006) and LP-UFC 722 (this work), and completely closed for the subadult specimens THUg 1907 (Gaffney, Tong & Meylan, 2006) and MN 6949-V (this work). Taphonomic alteration is not a possible explanation for this diversity since different conditions can be seen in excellent three-dimensionally preserved skulls from the Romualdo Fm. that exhibit undamaged surfaces of the exoccipital (Gaffney, Tong & Meylan, 2006). Indeed, Gaffney, Tong & Meylan (2006) considered this variation to exist and coded *A. barretoi* as polymorphic for this character in their analysis. This feature cannot be regarded as sexually dimorphic because the same condition (open) can be seen in AMNH 24453 (a female) and in LP-UFC 722 (a male). Finally, as Gaffney, Tong & Meylan (2006) had already pointed out, it could not be explained by ontogeny because different conditions can be seen in AMNH 24454 (open) and THUg 1907 (closed), which are both of similar size and close to osteological maturity (Gaffney, Tong & Meylan, 2006). Because THUg 1357 is a juvenile and exhibits an open foramen jugulare posterius, it remains unclear if this specimen would exhibit an open or closed configuration if it had achieved a subadult stage with more ossification (Gaffney, Tong & Meylan, 2006).

Another variation can be found in the shape of the paraoccipital process of the opisthotic, which has a convex posteromedial margin in THUg 1357 and THUg 1907, and a concave one in AMNH 24453 and AMNH 24454 (Fig. S6). Unfortunately, the condition is unclear for MN 6949-V and LP-UFC 722, in which the tips of the paraoccipital processes have been lost.

The mandible of specimens attributed to *A. barretoi* further provide a curious case of a repeated intra-individual variation, specifically in the coronoid. In three specimens (MN 6949-V, AMNH 24454 and THUg 1907), the left coronoid is considerably more developed than the right coronoid. In all three specimens, the right coronoid is relatively small, mostly restricted to the posterior half of the coronoid process. The anterior half is occupied mainly by the dentary, with the participation of the coronoid restricted to a slender dorsal projection on the anterodorsal margin. On the left hemimandible, however, the coronoid is relatively more extended anteriorly, occupying the anterior half of the coronoid process in AMNH 24454 and MN 6949-V and most of it in THUg 1907.

#### Neural bones

There is an astonishing amount of variation on neural bones throughout Testudines (Pritchard, 1988). The presumed ancestral condition of about 8 predominantly hexagonal neurals can be modified by proliferation, reduction and changes in shape of neurals (Pritchard, 2008). Variation on neural bones occur both between species and between individuals of the same species (Pritchard, 1988). *A. barretoi* has typically nine neural bones with the following neural formula 6 > 6 > 4 < 6 < 6 < 6 < 6 > 6 > 3. By constrast, MPSC R 010 has 10 neural bones instead of nine and the following neural formula 6 < 6 > 4 < 5 > 6 > 6 > 6 > 6 > 4 < 4. It deviates from the typical pattern of *A. barretoi* by having neural 1 smaller than neural 2, neural 4 reaches left costal 3 and, besides an additional element at the end of series, the last neural has four contacts instead of three. In UFRPE 5302, most of the neural bones were lost given the specimen is only a fragment of a shell. Nevertheless, this specimen shows two deviations from the typical shell morphology of *A. barretoi*: the neural series is interrupted at the end by the pair of costals 8, which meet at midline, thus precluding the contact between neural 9 and the suprapygal (Fig. S1; Fig. 5). This condition has already been reported for *A. barretoi* specimens from Romualdo Fm. by Meylan (1996; specimens AMNH 22556 and AMNH 24453), and by Schleich (1990; specimen BSP 1981 I 38). The second deviation is the pygal bone, which has a triangular shape instead of the usual square shape, and do not contact the suprapygal, with peripherals 11 from both sides meeting at the midline precluding the contact between the pygal and suprapygal (Fig. S1). The specimen MN 6744-V also exhibits contact between the pair of costals 8, and is further variable in exhibiting a rounded last neural, like BSP 1981 I 38.

In this way, there are, among our specimens with complete neural series, seven specimens with 10 neurals (DGM 756-R, MPSC R 134, MPSC R 874, MPSC R 2308, MPSC R 010, MN 6949-V, MN 7191-V), six specimens with nine neurals (MN 6743-V, MN 6744-V, AMNH 24453, AMNH 22550, AMNH 22556, BSP 1981 I 38) and one specimen with eight neurals (LP-UFC 722), other than an incomplete specimen with, most likely, eight neurals as well (UFRPE 5302). There are several specimens with an incomplete neural series and it is thus unclear what configuration is more common, and could be considered ‘typical’ for *A. barretoi*. Nonetheless, the condition of eight neurals, similar to *T. decorata*, seems to be the least common.

The last neural contacts the suprapygal in at least five specimens (LP-UFC 722, AMNH 22550, MPSC R 010, MPSC R 874, MN 6949-V) and does not in five specimens (UFRPE 5302, MN 6744-V, AMNH 22556, AMNH 24453 and BSP 1981 I 38).

A rounded last neural can be found in three specimens (DGM 756-R, MN 6744-V, BSP 1981 I 38), and a triangular pygal that does not contact the suprapygal is so far reported exclusively for UFRPE 5302. In this way, we consider these two features to most likely represent variations from the typical pattern: a polygonal last neural and the presence of a pygal–suprapygal contact.

#### Shape of the carapace

Another variation identified herein concerns the shape of the carapace in dorsal view. The holotype (Price, 1973), as well as the carapaces described by Meylan (1996), all exhibit a ‘squared’ morphology, that is, they exhibit a posterior extension as opposed to terminating in a gentle rounded curve. This characteristic has been considered as diagnostic for *A. barretoi* in the revised diagnosis of Sereno & ElShafie (2013) and has been used to distinguish that species from the purported species *A*. ‘*arturi’* by Fielding, Martill & Naish (2005), whose holotype (SMNK PAL 3979) exhibits an oval-shaped carapace. We found the oval-shaped morphology in the specimens LP-UFC 722, DGM 346-LE, MN 6744-V and MPSC R 134, other than the previously described specimen BSP 1981 I 38 (Schleich, 1990). The square-shaped morphology can be seen in MN 6949-V, DGM 1449-R, MPSC R 010, MPSC R 874, MPSC R 135 and UFRPE 5302, other than the previously reported specimens AMNH 24453, AMNH 22550, AMNH 22556 (Meylan, 1996) and the SMNK’s postcranial skeleton (Naish, 2007). It is thus likely that the typical configuration is the square-shaped morphology, with the oval morphology as a variation. This individual variation is interpreted here as a polymorphism, and not related to sexual dimorphism, because LP-UFC 722 and MN 6949-V are both inferred males, notwithstanding exhibiting distinct carapace shapes. In the same way, AMNH 24453 is an inferred female specimen that exhibits a carapace shape similar to MN 6949-V.

Furthermore, there also exists variation in the region of carapacial expansion within the square-shaped carapaces. In the holotype, MN 6949-V, MPSC R 134 and BSP 1981 I 38, the most acute angulation in the carapace, which indicates the lateral expansion, is present between peripherals 8 and 9. In specimens DGM 1449-R, AMNH 22550 and AMNH 22553, however, the lateral expansion is more prominent between peripherals 9 and 10. In specimens UFRPE 5302, MPSC R 137 and MSPC R 010, the most acute angle in formed between peripherals 7 and 8, similar to *T. decorata* (Sereno & ElShafie, 2013).

#### Shell texture

Shell ornamentation has been for long used as a character in turtle identification and phylogeny (De la Fuente & Lapparent de Broin, 1997; Gaffney, Tong & Meylan, 2006; Sereno & ElShafie, 2013). Characters related to shell surface texture grow in importance in palaeontology when describing fragmentary material (a common occurrence in the fossil record). Groups known by a constant and distinct ornamentation pattern are, for instance, the Trionychidae, whose members possess on their shells a unique surface ornamentation and subsurface structure (Scheyer et al., 2007) allowing ‘even the smallest fossil fragment’ to be recognised in the fossil record (Joyce & Lyson, 2010). Also for pleurodires, many authors have resorted to ornamentation patterns in order to identify their specimens, or when diagnosing species or constructing phylogenetic hypothesis (see a review in Gaffney, Tong & Meylan, 2006). A ‘pelomedusoid’ ornamentation (‘a pattern of reticulate and anastomosing furrows and/or long striations that do not radiate from growth centers’; Gaffney, Tong & Meylan, 2006) was recognised and described by Lapparent de Broin (1977; ‘décoration pélomédusidienne’). Araripemydidae (sensu Sereno & ElShafie, 2013) is supposed to be characterised by a distinctive ornamentation pitted pattern, as can be seen from the character 175 by Gaffney, Tong & Meylan (2006), ‘shell texture’, with state five being ‘pits (*A. barretoi*)’.

To this date, two fragmentary turtles were referred as related to Araripemydidae based on the ornamentation pattern. Lapparent de Broin (1980) described a new genus and species, *T. decorata*, from the Elrhaz Fm. (Niger); later, De la Fuente & Lapparent de Broin (1997) tentatively referred a specimen from the Palaeocene of Argentina as an *Araripemys*-like taxon. Both descriptions relied heavily on the distinctive pitted ornamentation displayed by the specimens, considered typical of *A. barretoi*. It is worth mentioning that the holotype specimen of *Laganemis tenerensis* was originally referred to the genus *Araripemys*, when first presented to the scientific community (Sereno & ElShafie, 2013). Recently, *L. tenerensis* was recognised as a junior synonym of *T. decorata* by Pérez-García (2018). The synonymisation was based on—among other characters-variation of the shell ornamentation already reported by Lapparent de Broin (1980). Pérez-García (2018) considered this variation as of intraspecific nature.

MPSC R 010 exhibits the pitted ornamentation on several costals and neurals of the carapace, and on every bone of the plastron, but it shows no signs of the ridge-and-sulcus ornamentation. UFRPE 5302 has the typical pitted ornamentation covering densely the suprapygal, all the costals and neurals, while the peripherals exhibit the ridge-and-sulcus ornamentation instead. The carapace surface texture of LP-UFC 722 is mostly unknown due to the dorsal decubitus of the specimen. However, both the pitted and ridge-and-sulcus ornamentation can be seen on the peripherals, contrasting with MPSC R 010, which has no ornamentation whatsoever on the peripherals (similar to BSP 1981 I 38 and AMNH 22550), and with UFRPE 5302, which has the ridge-and-sulcus ornamentation on the peripherals instead of the pitted one. The plastron of LP-UFC 722 exhibits both types of ornamentation: the pitted ornamentation heavily on the epiplastra and entoplastron, and slightly on all other bones, while the hyo- and hypoplastra display the ridge-and-sulcus ornamentation—similarly to AMNH 24453. MN 6949-V includes all the shell elements preserved, and all of them exhibit exclusively the pitted pattern (Fig. 7). The same is true for DGM 346-LE (despite the incomplete surface of the carapace; pits can be seen in costals and peripherals), MN 6744-V (with only the carapace visible, costals and peripherals) and DGM 1449-R (only a few carapace elements with preserved surfaces; neurals, peripherals and costals; Fig. 7).

In this way, we consider as variations of the extensive pitted pattern the following configurations: smooth peripherals (MPSC R 010, MPSC R 137, AMNH 22550 and BSP 1981 I 38), ridge-and-sulcus pattern on peripherals (UFRPE 5302), combined pitted and rigde-and-sulcus ornamentation on peripherals (LP-UFC 722 and DGM 1449-R) and ridge-and-sulcus pattern on the hyo-, hypo- and xiphiplastra, but not on entoplastron and epiplastra (LP-UFC 722, AMNH 24453, MPSC R 2107 and MN 6743-V).

### Unguals

The unguals of *A. barretoi* were described for the first time by Meylan (1996), based on the specimens AMNH 24453, 24454, 24455 and 22550. In the combined reconstruction, the unguals are figured as arrowhead-shaped, to the exception of manual digit V, which is simple; the ungual of pedal digit V was unknown. Subsequently, Fielding, Martill & Naish (2005) described the specimen SMNK-PAL 3979 (holotype of *A*. ‘*arturi’*), which exhibits simple unguals instead of arrowhead-shaped ones (Fig. S7), and interpreted the shape differences as a taxonomic feature distinguishing *A*. ‘*arturi’* from *A. barretoi*. Later on, Oliveira & Kellner (2007) figured the manual unguals of MN 6949-V, which are also simple, considering thus *A*. ‘*arturi’* as a junior synonym of *A. barretoi*. More recently, Oliveira & Kellner (2017) reported two Crato Fm. hatchling specimens, MN 4893-V and AMNH 30651, both exhibiting arrowhead-shaped unguals. These specimens were attributed to *Araripemys* cf. *A. barretoi* because of, among other characters, the arrowhead-shaped unguals.

Our comparative sample comprises 11 specimens for which the unguals are known, including some of the illustrated described here (LP-UFC 722, MN 6949-V, MN 6637-V) as well as some of the comparative specimens (AMNH numbers: 24453, 24454, 24455, 22550, 30651; SMNK-PAL 3979, MN 4893-V). This variation seems not to be correlated to sex or ontogenetic stage.

### Ontogeny: intercostal fontanelles

Intercostals fontanelles could be, potentially, morphological features associated with sexual dimorphism. The presence of intercostal fontanelles in adults is characteristic for males of some extant species for example *Macrochelys temminckii*, and for females of some other species for example *Graptemys barbouri* (Pritchard, 2008). However, in our sample, MPSC R 010, a female, also displays intercostal fontanelles. As intercostal fontanelles were found in all *A. barretoi* specimens herein and previously described, comprising the putative two sexes among the specimens of this study, this feature is rendered inconclusive for sexual determination in *A. barretoi*.

Costal fontanelles are, on the other hand, often present as juvenile features in many extant taxa, especially among chelids. In other instances, the persistence of large carapacial fontanelles could be related to cases of paedomorphosis (Li et al., 2008; Reisz & Head, 2008). All described specimens of *A. barretoi* exhibit costal fontanelles (contrasting with *T. decorata*, which lacks costal fontanelles; Sereno & ElShafie, 2013); notwithstanding, variation concerning their relative sizes were observed.

The hatchling specimen AMNH 30651 exhibits thin, slender, completely unfused costals and peripherals were not even ossified yet (Oliveira & Kellner, 2017). In the small, early juvenile specimen MPSC R 137 (150 cm in length × 140 cm in width), the costals contact each other only in the medial half, with large fontanelles enclosed between them and the peripherals. In the larger, presumably subadult specimens MN 6949-V, MPSC R 010 and UFRPE 5302, contact between costals extends further laterally and the fontanelles become relatively smaller. Finally, the large specimens MPSC R 134 and DGM 1449-R exhibit comparatively diminutive costal fontanelles. This variation is thus here regarded as ontogenetic and presumably related to the ossification of the costal bones, increasing with age and diminishing the relative size of the intercostal fontanelles.

### Problematic, co-occurring features: potential interspecific variations?

Some of the variations here reported are difficult to regard with much certainty as, indeed, intraspecific variations of any sort—either polymorphism, anomalies or sexual dimorphism—or ontogenetic changes. For instance, the condition of the foramen jugulare posterius which varies from completely open to completely closed in *A. barretoi* (Gaffney, Tong & Meylan, 2006). As described above, the following conditions are known: open for the juvenile specimen THUg 1357 and the subadult specimens AMNH 24453, AMNH 24454 (Gaffney, Tong & Meylan, 2006) and LP-UFC 722 (this work), and completely closed for the subadult specimens THUg 1907 (Gaffney, Tong & Meylan, 2006) and MN 6949-V (this work). Its interpretation as a polymorphism is severely hampered by the fact that such variation is unseen in any pleurodire species (Gaffney, Tong & Meylan, 2006; p. 142, first column, lines 45–48). We thus propose here the existence of two morphotypes of *A. barretoi* in the Araripe Basin. For practical reasons, we shall now refer as morphotype 1 the one that exhibits an open condition and morphotype 2 the one with the closed condition. Because THUg 1357 is a juvenile and exhibits an open foramen jugulare posterius, it remains unclear if this specimen would exhibit an open or closed configuration if it had achieved a subadult stage with more ossification (Gaffney, Tong & Meylan, 2006). The condition in this specimen is not as widely open as it is in AMNH 24453 or AMNH 24454, existing a subtle ventral and lateral expansion of the opisthotic above it, so that it is possible that, had it grown further, it would have developed an enclosed foramen (Gaffney, Tong & Meylan, 2006).

Secondly, the shape of the ungual is also problematic. Arrow-head shaped unguals have been regarded as diagnostic of *A. barretoi* (Sereno & ElShafie, 2013; Oliveira & Kellner, 2017) subsequently to the description of AMNH 24453 and AMNH 24454 by Meylan (1996). The holotype of *A*. ‘*arturi’* displays simple unguals, what led Fielding, Martill & Naish (2005) to regard this feature as diagnostic of the proposed new species, differentiating it from *A. barretoi*, among other features. As mentioned before, these two species have been synonymised under *A. barretoi* and their differences regarded as intraspecific (Gaffney, Tong & Meylan, 2006; Oliveira & Kellner, 2007). However, together with the condition of the foramen jugulare posterius, we completely ignore the existence of any turtle species displaying such a degree of intraspecific variation in the shape of the unguals. In addition, regarding our sample as currently known (though acknowledgedly limited as it may be), this variation coincides with the variation in the foramen jugulare posterius. Arrow-head shaped unguals are present in specimens AMNH 24453, AMNH 24454 and LP-UFC 722, which exhibit an open foramen jugulare posterius; while the unguals are simple in specimen MN 6949-V, which displays a closed foramen jugulare posterius. In this way, morphotype 1 would be characterised by an open foramen jugulare posterius and arrow-head shaped unguals, and morphotype 2 by a closed foramen jugulare posterius and simple unguals.

Thirdly, these conditions further coincide with the plastral ornamentation pattern (Table S1). The ornamentation is pitted in the costals, neurals, nuchal and pygal series, as well as on the epiplastra and entoplastra, in every specimen where these elements are visible. However, the ornamentation can consist of a sulcus-and-ridge pattern in the hyo-, hypo- and xiphiplastra in some specimens, while pitted in others. Specimens LP-UFC 722 and AMNH 24453 exhibit an open foramen jugulare posterius, arrow-head shaped unguals and ridge-and-sulcus ornamentation on the hyo-, hypo- and xiphiplastra. On the other hand, specimen MN 6949-V exhibits a closed foramen jugulare posterius, simple unguals and pitted ornamentation on all shell elements. Specimens SMNK PAL 3979 exhibits a pitted ornamentation on the hyo- and hypoplastra also, as well as simple unguals (skull unknown). Specimen BSP 1977 I 1 exhibits a pitted ornamentation on all plastral elements and simple unguals as well (skull unknown).

There also seems to be a co-occurring variation involving the shape of the paraoccipital process of the opisthotic. As pointed out, the juvenile specimen THUg 1357 and the subadult specimen THUg 1907 exhibit a convex medial margin of this process, while AMNH 24453 and AMNH 24454 clearly exhibit a concave margin. This difference could also be related to the morphotypic pattern, with the concave margin coinciding with the morphotype 1, while the convex margin coincides with morphotype 2. However, we refrain from affirming this with certainty before more well-preserved skulls are described, since the tips of the paraoccipital processes of MN 6949-V and LP-UFC 722 are broken off, precluding confirmation of their shapes.

Lastly, AMNH 24454 and LP-UFC 722 share a unique feature, which is the ‘extra foramen’ described by Gaffney, Tong & Meylan (2006) for the former specimen. It is located in the suture between the exoccipitals and opisthotics, at the base of the paraoccipital processes. This foramen is lacking completely in MN 6949-V, THUg 1357 and THUg 1907 (undescribed for AMNH 24453).

These comparisons suggest that morphotype 1 could likely be characterised by (1) an open foramen jugulare posterius, (2) arrow-head shaped unguals, (3) the presence of ridge-and-sulcus ornamentation in the hyo-, hypo- and xiphiplastra, and possibly (4) presence of an extra foramen on the suture between exoccipitals and opisthotics. Morphotype 2, in turn, would be characterised by (1) a closed foramen jugulare posterius, (2) simple unguals, and (3) pitted ornamentation on all plastral elements. As a comparison, *T. decorata* exhibits a third, distinct combination: (1) an open foramen jugulare posterius, (2) simple unguals, and (3) ridge-and-sulcus ornamentation on all carapacial and plastral elements (Sereno & ElShafie, 2013).

The consistency of these morphotypes is to be confirmed, or not, by the detailed description of more specimens in the future, particularly of those housed in AMNH and THUg collections

## Conclusions

*Araripemys barretoi* is a remarkable fossil taxon in bearing so many individuals. Including the specimens described herein, there have been a total of 45 specimens referred to *A. barretoi* in the literature, to our knowledge (Price, 1973; Kischlat & de Almeida Campos, 1990; Schleich, 1990; Meylan, 1996; Fielding, Martill & Naish, 2005; Oliveira & Kellner, 2005; Gaffney, Tong & Meylan, 2006; Batista & Carvalho, 2007; Carvalho, Ghilardi & Barreto, 2016; Oliveira & Kellner, 2017; this work). This has allowed us to compile a particularly detailed account of the morphological variation seen in a fossil turtle.

Our identification of two morphotypes characterised by variations unreported as intraspecific variations within any known species provides evidence for the possibility that what is currently known as *A. barretoi* is actually composed of two distinct species. In such a scenario, morphotype 1 would represent a new species, while morphotype 2 would correspond to the type species *A. barretoi* and *A*. ‘*arturi’* as well as a junior synonym.

However, given our relatively low skull sample (which is still rather high for a fossil species), we refrain from affirming with confidence the existence of two species of *Araripemys* before more material can corroborate this hypothesis. A geometric morphometric analysis of the skull is also planned in the future development of this investigation, as a mean of further testing this hypothesis.

We finish by quoting Gaffney, Tong & Meylan (2006) who, in a memorable line in their paper, when trying to explain the meaning of the complex variation exhibited by the foramen jugulare posterius of *A. barretoi*, put plainly (but apparently not without some exasperation): ‘It all goes to show that some of this crap makes no sense at all’ (Gaffney, Tong & Meylan, 2006; p. 148, line 53). We hope we have helped to disentangle this issue a bit.

## Supplemental Information

10.7717/peerj.9840/supp-1 Supplemental Information 1 Variations and sizes of studied specimens. For each specimen, the following information is summarised in a comparative table: ornamentation of shell bones; carapace and plastron length and width; number of neural bones; presence or absence of neural - suprapygal contact; neural formula; ungual morphology; inferred sex; geological formation where the fossil was retrieved.

10.7717/peerj.9840/supp-2 Supplemental Information 2 Detailed osteological description of all figured specimens. Every single Araripemys specimens discussed and figured in the main file are here described (or redescribed) in detail one by one.

10.7717/peerj.9840/supp-3 Supplemental Information 3 Specimens MPSC-2107 and UFRPE-5302. A) MPSC R 2107, plastron in ventral view and B) schematic drawing. Photo and drawing by Thales Nascimento. C) UFRPE 5302, carapace in dorsal view and D) schematic drawing. Photo and drawing by Thales Nascimento. Abbreviations: Ab – abdominal scute, An – anal scute, co – costal, Fe – femoral scute, Fen – fenestra, Hu – humeral scutes, hyo – hyoplastron, hypo – hypoplastron, Ma – marginal, ne – neural, Pe – peripherals, Pl – pleural scute, Py – pygal, Spy – suprapygal, Ve – vertebral scute, Xip – xiphiplastron.

10.7717/peerj.9840/supp-4 Supplemental Information 4 Shells of several specimens – part I. Holotype (DGM 756-R), A) carapace, dorsal view and B) plastron, ventral view. Photo by Rodrigo V. Pêgas. MPSC R 2308, C) carapace in dorsal view and D) plastron in ventral view. Photo by Renan Bantim. DGM 346-LE. E), F), G), and H), respective schematic drawings. I) Carapace in dorsal view and J) plastron in ventral view. Photo by Rodrigo V. Pêgas and Thales Nascimento. MN 6744-V, K) part with carapace in dorsal view and L) counterpart with carapace remains in internal view. M), N), O) and P), respective schematic drawings. Photo by Rodrigo V. Pêgas and Thales Nascimento. Abbreviations: car – carapace, co – costal, en – entoplastron, ep – epiplastron, fe – femur, Hu – humeral scute, hyo – hyoplastron, hyp – hypoplastron, Ma – marginal, n – neural, nu – nuchal, pe – peripheral, Pl – pleural, py – pygal, spy – suprapygal, xip – xhiphiplastron.

10.7717/peerj.9840/supp-5 Supplemental Information 5 Shells of several specimens – part II. BSP 1977 I 1, A) carapace and B) plastron. Photo by Rodrigo V. Pêgas. BSP 1981 I 38, C) carapace and D) plastron. Photo by Rodrigo V. Pêgas. E), F), G), H), respective schematic drawings. MN 6637-V. I) carapace, encased in resin and J) plastron. Photo by Rodrigo V. Pêgas and Thales Nascimento. MN 6743-V. K) Impression of the carapace in internal view and L) plastron in visceral view. Photo by Rodrigo V. Pêgas and Thales Nascimento. M), N), O) and P), respective schematic drawings. Abbreviations: Ab – abdominal scute, co – costal, en – entoplastron, ep – epiplastron, Fe – femoral scute, fe – femur, Hu – humeral scute, hu – humerus, hyo – hyoplastron, hypo – hypoplastron, n – neural, nu – nuchal, pe – peripheral, py – pygal, ra – radius, rb – rib, spy – suprapygal, Ve – vertebral scute, xip – xhiphiplastron.

10.7717/peerj.9840/supp-6 Supplemental Information 6 Carapaces in dorsal view. A) and B), DGM 1449-R. Photo by Rodrigo V. Pêgas. C) and D) MPSC R 134. Photo by Renan Bantim. E) and F) MPSC R 137. Photos by Renan Bantim. G) and H) SMNK no number. Photo by Rodrigo V. Pêgas. Abbreviations: co – costal, Ma – marginal scute, n – neural, nu – nuchal, pe – peripheral, py – pygal, spy – suprapygal.

10.7717/peerj.9840/supp-7 Supplemental Information 7 Example of skull variation seen in the extant *Chelonia mydas*.. A), male, presenting contact between prefrontals and postorbitals. B), female with frontals separating prefrontals from the postorbitals and reaching the orbit margin.

10.7717/peerj.9840/supp-8 Supplemental Information 8 Conditions of the opisthotic. A) convex in THUg 1357 and B) concave in AMNH 24453.

10.7717/peerj.9840/supp-9 Supplemental Information 9 SMNK PAL 3979. A) Whole specimen, ventral view. B) Plastral elements showing pitted ornamentation on the preserved ventral surface. C) Pes showing simple unguals. Photos by Rodrigo V. Pêgas.

## Institutional Abbreviations

AMNH: American Museum of Natural History (New York, United States)
BSP: Bayerischen Staatssammlung für Paläontologie und Geologie (Munich, Germany)
DGM/DNPM: Divisão de Geologia e Mineralogia, Museu de Ciências da Terra, Departamento Nacional da Produção Mineral (Rio de Janeiro, Brazil)
LP-UFC: Laboratório de Paleontologia, Universidade Federal do Ceará (Fortaleza, Brazil)
MN: Museu Nacional, Universidade Federal do Rio de Janeiro (Rio de Janeiro, Brazil)
MPSC: Museu de Paleontologia de Santana do Cariri (Santana do Cariri, Brazil)
UFRPE: Universidade Federal Rural de Pernambuco (Recife, Brazil)
SNM: Statens Naturhistoriske Museum (Copenhagen, Denmark)
SMNK: Staatliches Museum für Naturkunde, Karlsruhe (Karlsruhe, Germany)
THUg: Teikyo Heisey University (Chiba, Japan)

## Anatomical Abbreviations

Ab: abdominal scute
An: anal scute
an: angular
ar: articular
bo: basioccipital
bs: basisphenoid
c: cervical vertebrae
car: carapace
cdv: caudal vertebrae
co: costal
cor: coronoid
d: dentary
dg: digit
dv: dorsal vertebrae
ef: extra foramen
en: entoplastron
ep: epiplastron
ex: exoccipital
Fe: femoral scute
fe: femur
fjp: foramen jugulare posterius
fon: fontanelle
fpcci: foramen posterius canalis carotici interni
fr: frontal
fst: foramen stapedio temporale
Hu: humeral scute
hu: humerus
hy: hyoid
hyo: hyoplastron
hyp: hypoplastron
isc: ischium
ju: jugal
lj: lower jaw
Ma: marginal scute
mc: metacarpal
mx: maxillary
ne: neural
op: opisthotic
p: peripherals
pa: parietal
pal: palatine
Pe: pectoral scute
pf: prefrontal
ph: phalanx
Pl: pleural scute
pmx: premaxilla
po: postorbital
pr: prootic
pt: pterygoid
py: pygal
q: quadrate
qj: quadratojugal
ra: radius
rb: rib
sa: surangular
soc: supraoccipital
spy: suprapygal
sq: squamosal
st: stapes
ul: ulna
un: ungual
Ve: vertebral scute
xp: xiphiplastron.

## Additional Information and Declarations

### Data Availability

The following information was supplied regarding data availability: Raw data is available at Figshare: Limaverde, Saulo; Pêgas, Rodrigo Vargas; Villa, Chiara; Oliveira, Gustavo Ribeiro De; Bonde, Niels; de Castro Leal, Maria Eduarda (2020): LP-UFC 722 CT Scans. figshare. Dataset. DOI 10.6084/m9.figshare.7700138.v1. The data is also available at MorphoSource: https://www.morphosource.org/Detail/ProjectDetail/Show/project_id/989. - Specimen: UFC:LP:722, Araripemys barretoi, Media: M62994, https://www.morphosource.org/Detail/SpecimenDetail/show/specimen_id/30412. - Specimen: UFC:LP:722, Araripemys barretoi, Media: M65270, https://www.morphosource.org/Detail/MediaDetail/Show/media_id/65270. List of specimens and institutions: - Laboratório de Paleontologia, Universidade Federal do Ceará (Fortaleza, Brazil), LP-UFC 722; - Museu de Paleontologia de Santana do Cariri (Santana do Cariri, Brazil), MPSC R 010; - Museu de Paleontologia de Santana do Cariri (Santana do Cariri, Brazil), MPSC R 2107; - Museu de Paleontologia de Santana do Cariri (Santana do Cariri, Brazil), MPSC R 2308; - Museu de Paleontologia de Santana do Cariri (Santana do Cariri, Brazil), MPSC R 874; - Museu de Paleontologia de Santana do Cariri (Santana do Cariri, Brazil), MPSC R 134; - Museu de Paleontologia de Santana do Cariri (Santana do Cariri, Brazil), MPSC R 137; - Museu Nacional, Universidade Federal do Rio de Janeiro (Rio de Janeiro, Brazil), MN 6949-V*; - Museu Nacional, Universidade Federal do Rio de Janeiro (Rio de Janeiro, Brazil), MN 6743-V; - Museu Nacional, Universidade Federal do Rio de Janeiro (Rio de Janeiro, Brazil), MN 6744-V; - Museu Nacional, Universidade Federal do Rio de Janeiro (Rio de Janeiro, Brazil), MN 6637-V; - Divisão de Geologia e Mineralogia, Museu de Ciências da Terra, Departamento Nacional da - Produção Mineral (Rio de Janeiro, Brazil), DGM 346-LE; - Divisão de Geologia e Mineralogia, Museu de Ciências da Terra, Departamento Nacional da - Produção Mineral (Rio de Janeiro, Brazil), DGM 1449-R; - Divisão de Geologia e Mineralogia, Museu de Ciências da Terra, Departamento Nacional da - Produção Mineral (Rio de Janeiro, Brazil), DGM 756-R; - Staatliches Museum für Naturkunde, Karlsruhe (Karlsruhe, Germany), SMNK PAL 3979; - Staatliches Museum für Naturkunde, Karlsruhe (Karlsruhe, Germany), SMNK PAL (no number); - Bayerischen Staatssammlung für Paläontologie und Geologie (Munich, Germany), BSP 1977 I 1; - Bayerischen Staatssammlung für Paläontologie und Geologie (Munich, Germany), BSP 1981 I 38; - (Universidade Federal Rural de Pernambuco (Recife, Brazil), UFRPE 5302. * The specimen MN 6949-V was lost during a fire in the Brazilian National Museum in the night of September 2nd 2018.
