# Supplementary material for: Interpreting character variation in turtles: Araripemys barretoi (Pleurodira: Pelomedusoides) from the Araripe Basin, Early Cretaceous of Northeastern Brazil
Source: PeerJ. 2020 Sep 29;8:e9840. doi: 10.7717/peerj.9840 (PMC7531347; doi:10.7717/peerj.9840)
Supplement: Supplemental Information 1 — For each specimen, the following information is summarised in a comparative table: ornamentation of shell bones; carapace and plastron length and width; number of neural bones; presence or absence of neural - suprapygal contact; neural formula; ungual morphology; inferred sex; geological formation where the fossil was retrieved. [file peerj-08-9840-s001.docx]

**The challenges of interpreting character variation in turtles: *Araripemys barretoi* (Pleurodira: Pelomedusoides) from the Araripe Basin, Early Cretaceous of Northeastern Brazil**

Saulo Limaverde, Rodrigo V. Pêgas, Rafael C. Damasceno, Chiara Villa, Gustavo R. Oliveira, Niels Bonde, Maria Eduarda C. Leal

**SUPPLEMENTARY MATERIAL 2**

**Comparative Table S1**

|  | Specimen | Costals, neurals, suprapygal | Plastral bones | Peripherals | Carapace length  x width (mm) | Plastron length  x width (mm) | Number of  neurals | Neural-suprapygal contact | Neural Formula | Ungual shape | Inferred sex | Formation |
| --- | --- | --- | --- | --- | --- | --- | --- | --- | --- | --- | --- | --- |
| 0 | *Taquetochelys decorata* | Sulcus and pits | Sulcus and pits | Sulcus and pits | 144 x 140 | 130 x 101 | 8 | No | 6 > 6 > 4 < 6 < 6 < 6 < 6 > 5 | Simple | ? | Elrhaz |
| 1 | Holotype (DGM 756-R) | Pits | Pits | Pits | ? x 221,7 | ? x 213 | 10 | Yes | 6 < 6 > 4 = 6 > 6 > 6 > 6 > 6 > 4 > 3 | ? | Male | Romualdo |
| 2 | MN 6743-V | Pits | Sulcus + pits | Pits | 138 x 116 | ? | 9 | Yes | ? – 6 < 4 > 6 < 6 = 6 > 6 > 4 > 3 | ? | ? | Romualdo |
| 3 | MN 6637-V | Pits | ? | ? | ? | ? | 9 | ? | ? | Arrow | Male | Romualdo |
| 4 | MN 6744-V | Pits | ? | Pits | 150 x 150 | ? | 9 | No | 6 > 6 < 4 > 6 > 6 > 6 > 6 > 4 > 3 | ? | ? | Romualdo |
| 5 | MN 6949-V | Pits | Pits | Pits | 165 x 140 | 132 x 136 | 10 | Yes | 6 = 6 < 4 < 5 > 6 > 6 > 6 > 6 > 4 > 4 | Simple | Male | Romualdo |
| 6 | MN 7191-V | Pits | ? | ? | 155 x 127 | ? | 10 | Yes | ? | ? | ? | Romualdo |
| 7 | DGM 1449-R | Pits | ? | Sulcus + pits | 223 x 225 | ? | ?9 | ?No | ? | ? | ? | Romualdo |
| 8 | DGM 364-LE | Pits | Pits | Pits | 210 x 167 | 173 x 162 | ? |  | ? | ? | ? | Romualdo |
| 9 | SMNK-PAL 3979 | ? | Pits | Pits | 180 x ? | ? | ? | ? | ? | Simple | ? | Crato |
| 10 | LP-UFC 722 | ? | Sulcus + pits | Sulcus + pits | 185 x 200 | 160 x 175 | 8 | ? | ? – 6 > 4 < 6 < 6 - ? - ? – 4 | Arrow | Male | Crato |
| 11 | MPSC R 010 | Pits | Pits | Pits | 140 x 106 | 100 x 100 | 10 | Yes | 6 < 6 > 4 < 5 > 6 > 6 > 6 > 6 > 4 < 4 | ?Simple | Female | Romualdo |
| 12 | MPSC 2107 | ? | Sulcus | ? | ? | 150 x 150 | ? | ? | ? | ? | Male | Crato |
| 13 | UFRPE 5302 | Pits | ? | Sulcus | 130 x ? | ? | ?8 | No | ? | ? | ? | Crato |
| 14 | MPSC R 134 | Pits | ? | Pits | 250 x 250 | ? | 10 | Yes | ? | ? | ? | Romualdo |
| 15 | MPSC R 137 | Pits | ? | Smooth | 150 x 140 | ? | ?9 | ?Yes | ? | ? | ? | Romualdo |
| 16 | MPSC R 874 | Pits | ? | Pits | 190 x 210 | ? | 10 | Yes | ? | ? | Male | Romualdo |
| 17 | MPSC R 2308 | Pits | Pits | Pits | 190 x 173 | ? | 10 | Yes | ? | ? | Male | Romualdo |
| 18 | AMNH 24452 | Pits | Pits | ? | ? | ? | ?10 | ?Yes | ? | ? | ? | Romualdo |
| 19 | AMNH 24453 | Pits | Sulcus | ? | ? | ? | 9 | No | 6 > 5 > 5 < 6 < 6 < 6 < 6 > 6 > 3 | Arrow | ? | Romualdo |
| 20 | AMNH 24454 | ? | ? | ? | ? | ? | ? | ? | ? | Arrow | ? | Romualdo |
| 21 | AMNH 22550 | ? | ? | ? | ? | ? | 9 | Yes | 6 > 6 > 4 < 6 < 6 < 6 < 6 > 6 > 3 | ? | ? | Romualdo |
| 22 | AMNH 22556 | ? | ? | ? | ? | ? | 9 | No | 6 > 6 > 4 < 6 < 6 < 6 < 6 > 6 > 3 | ? | ? | Romualdo |
| 23 | AMNH 22555 | ? | ? | ? | ? | ? | ? | ? | ? | Arrow | ? | Romualdo |
| 24 | BSP 1981 I 38 | Pits | Pits | Pits | 221 x 190 | ? x 156 | 9 | No | 6 < 6 > 5 < 6 > 6 > 6 > 6 > 6 > 3 | ? | Female | Romualdo |
| 25 | BSP 1977 I 1 | Pits | Pits | Pits | 215 x 180 | 180 x 136 | 10 | Yes | 6 < 6 > 4 < 6 > 6 = 6 > 6 > 4 > 3 | Simple | Male | Romualdo |
| 28 | UFPE 6773 | Pits | ? | Pits | (3 = 1,8) 9,8 x 10,4 | ? | ? | Yes | ? | ? | ? | Romualdo |
| 29 | SMNK n/n | Pits | ? | Pits | 200 X ((11,5 * 5) / 3) | ? | 10 | Yes | 6 < 6 > 4 = 6 > 6 > 6 > 6 > 6 > 4 > 3 | ? | ? | Romualdo |
| 30 | UFPE 7527 | Pits | ? | ? | 8,9 x 9,1 | ? | ? | ? | ? | ? | ? | Romualdo |

Table S1. Variations and sizes of studied specimens.
