# Supplementary material for: Interpreting character variation in turtles: Araripemys barretoi (Pleurodira: Pelomedusoides) from the Araripe Basin, Early Cretaceous of Northeastern Brazil
Source: PeerJ. 2020 Sep 29;8:e9840. doi: 10.7717/peerj.9840 (PMC7531347; doi:10.7717/peerj.9840)
Supplement: Supplemental Information 2 — Every single Araripemys specimens discussed and figured in the main file are here described (or redescribed) in detail one by one. [file peerj-08-9840-s002.docx]

**Interpreting character variation in turtles: *Araripemys barretoi* (Pleurodira: Pelomedusoides) from the Araripe Basin, Early Cretaceous of Northeastern Brazil**

Saulo Limaverde, Rodrigo V. Pêgas, Rafael C. Damasceno, Chiara Villa, Gustavo R. Oliveira, Niels Bonde, Maria E. C. Leal

**SUPPLEMENTARY MATERIAL**

**Detailed Descriptions**

In this Supplementary Material text we provide the detailed descriptions of all newly 14 reported as well the five revisited specimens. We refer the reader to the figures in the main part of the paper relative to each described specimen. In addition to those, please find also the Supplementary Figures S1 (relative to the vertebrae and axial elements of MN 6637-V) and S2 (the unprepared skull of the unnumbered Romualdo Formation SMNK specimen).

**New Specimens**

**LP-UFC 722**

This is an almost complete, articulated specimen exposed in ventral view, except for the skull, which has rotated at the atlas and lay in dorsoventral view and oriented caudally. LP-UFC 722 is preserved on the fine laminated cream-colored limestone from the Crato Fm., with most bones preserved tridimensionally. Flattening of the bones occurred only where hollow interiors were too weak to give support – the skull and the shell. It was retrieved in three separated slabs (parts a, b and c) which divide the specimen transversally at the height of the hyoplastra and longitudinally at the left side of the shell.

LP-UFC 722 presents a well-preserved skull, somewhat flattened but still largely tridimensional (Figs. 1-2); six cervical and nine dorsal vertebrae are visible, the dorsal vertebrae emerging partially through the plastron indicating the occurrence of diagenetic ventrodorsal flattening of the specimen. The right humerus is incomplete in the distal portion while the left humerus is complete, as well as the left radius and ulna. The left manus is exposed in lateral view. Both femora are preserved, as well as several other disarticulated hindlimb bones. The pair of ischia is exposed in lateral view and misplaced posteriorly to the xiphiplastra. The epiplastra are complete, except for the distal portion of the right epiplastron; entoplastra incomplete on the right distal portion; hyoplastra fragmented at the middle; hypoplastra and xiphiplastra present and complete; two fontanelles are visible, one between the hyoplastron and the hypoplastron and one between the hypoplastron and the xiphiplastron. The anterior fontanelle probably results from the breakage of the medial portion of the hyoplastron and originally composed two distinct fontanelles. Most of the caudal vertebrae are preserved, three of them in articulation (Fig. 2).

Some of the anatomic information for this specimen was retrieved through CT-Scan analysis. The files for the tomographic slices covering the entire specimen, the neural series, the head and neck and head-only can be found in the following link: [https://www.morphosource.org/Detail/ProjectDetail/Show/project_id/989](about:blank).

**Skull:** The skull is anteroposteriorly elongated, being about two times longer than wide. Due to diagenetic flattening, the skull roof collapsed in its midline near the contact between the frontals with each other and the parietals. The left parietal is misplaced to a higher position compared to the right parietal. The lower jaw is crushed against the skull and is slightly toggled to the right.

The orbits are anteroposteriorly elongated and lie close to each other dorsally in the skull along the nasal aperture. This is interpreted as a preservational artifact due to the diagenetic compressional collapse of the frontals at their suture line, making them lower than the other skull roof bones and bringing the orbits together at the skull midline.

The skull roof is composed by the frontals and parietals. Nasals are absent, as in other pelomedusoides (Meylan 1996; Gaffney *et al.*, 2006), and the prefrontals were not preserved. The supraoccipital crest extends posteriorly as far as the squamosal, and the processus paraoccipitalis formed by the opisthotic bone surpasses by far the supraoccipital crest, a feature of *A. barretoi*.

The premaxilla is a paired bone that contacts the maxillae posterolaterally and its counterpart medially. The only clearly visible sutures on the specimen are between the right premaxilla and maxilla and between both premaxillae. The pair of premaxillae forms the ventral margin of the nasal aperture and contributes a little to the labial ridge.

The maxillae contact anteriorly the premaxillae and, probably, the prefrontals as well - although the prefrontals are not preserved - the palatine medially and the jugal posteriorly forming the bulk of the upper jaw. It also composes the external margin of the nasal aperture and the ventral margin of the orbit. The maxillae and premaxillae surfaces are pierced by several diminutive neurovascular foramina.

The palatines form most of the fossa orbitalis. They are severely crushed on both sides and small pieces of it were lost during acid preparation. No suture could be asserted.

The jugals contact the maxillae anteroventrally through a very interdigitating suture and the postorbitals dorsally through an almost straight suture. They probably contacts the quadratojugals posterodosally as well, like all Pelomedusidae, Euraxemididae and most Bothremydidae (Gaffney *et al.,* 2006); however, in this specimen, the quadratojugal is displaced, and the coronoid covers the area where the contact is supposed to exist. The jugal constitutes a small portion of the posterior margin of the orbit and a large portion of the anterior margin of the cheek emargination.

The postorbitals are slender bones that run through the skull roof and contribute to the outline of both orbital margin and temporal emargination. They contact the frontals and parietals by its medial edge and the jugal anteriorly by most of its lateral edge. The contact with the quadratojugals cannot be seen.

The quadratojugals are a very slender bone that contacts the quadrates posteriorly. The right one is better preserved than its left counterpart, and is still contacting the quadrate but not the jugal nor the postorbital. Only the distal portion of the left quadratojugal can be seen standing against the left quadrate perpendicularly. By the relative position of the right quadratojugal it can be inferred that it constituted most of the cheek emargination.

The quadrates are a large bone from the posterior region of the skull bearing the cavum tympani. They contact the squamosals posterodorsally but not the opisthotics posteriorly, as expected in *A. barretoi*. Instead, it appears to contact the prootics which extends further posteriorly on this specimen precluding the contact between the two aforementioned bones. The quadrate also form part of the outline of the foramen stapediotemporale – which is clearly visible at the left side of the skull.

The squamosals contact the quadrates anteriorly and the opisthotics medially. The restoration of the *A. barretoi* skull presented in Gaffney *et al.*, (2006) shows that the squamosals contactsthe quadratojugals anteriorly through a very narrow suture, but this contact is not observed in this specimen since the quadratojugals are not in the original anatomical position in both sides of the skull.

The frontals are a pair of bones meeting each other at the midline, the postorbitals laterally and the parietals - with a strong interdigitating suture - posteriorly. The anterior edge constitutes the outline of the orbits, together with the postorbitals, jugals and maxillae.

The parietals comprise most of the skull roof. Anteriorly they contact the frontals along a very interdigitating suture. Medially they contact each other through a suture that is interdigitating in its first quarter and almost straight on the last three quarters. Laterally they contact the postorbitals. Posteriorly they contact the supraoccipital and possibly the prootic as well. The posterior margin is deeply embayed by the temporal emargination.

The prootic are preserved on both sides but their sutures cannot be clearly distinguished due to several brittle fractures running along the caudal portion of the skull.

In dorsal view, the opisthotics contact the squamosals and the exoccipitals laterally, it appears to reach the prootics anteriorly. It bears a proeminent process (processus paraoccipitalis) extending posteriorly beyond the squamosal, which is caractheristic of Pelomedusoides. The processus paraoccipitalis surpasses the supraoccipital crest in length. In the reconstruction based on the CT Scan images, it can be seen that the *foramen jugulare posterius* exhibits a completely open condition, similar to AMNH 24453 and AMNH 24454. In the opisthotic-exoccipital suture, a foramen is present, similar to AMNH 24454, termed an “extra foramen” by (Gaffney *et al.*, 2006).

The supraoccipital is almost entirely preserved except for a small portion of the crest just behind the dorsoanterior contact with the parietals. The other sutures (anterolaterally with the prootic and posterolaterally with the opistothic) could not be delimited.

The exoccipitals are preserved on both sides and comprise a large part of the foramen magnum – as expected for *A. barretoi*. Each exoccipital contacts the opisthotic laterally, but the sutures with the supraoccipital and basioccipital cannot be seen.

The basioccipital is presumably preserved since the condylus occipitalis is preserved, as seen on the CT scanning, and taking in account that the basioccipital contributes for a third of the condylus in *A. barretoi*. Nevertheless, none of the sutures could be seen.

The basisphenoids are not visible due to position of the skull in the matrix, but it could be seen on the CT scanning. The CT scanning reveals that the basisphenoid is preserved but it is mostly hidden by the left hyoid. No suture could be seen.

**Lower jaw:** The mandible is preserved almost in anatomical position. It is crushed beneath the skull, toggled to the right side. It is composed of the usual paired bones: dentary, coronoid, surangular, angular, prearticular and articular. The articular, angular, surangular, coronoid and dentary on the right jaw are fully exposed, but most of the left jaw lies underneath the skull and enclosed by the limestone matrix. The angulars are a slender bone that contacts the articular posteriorly, the surangular dorsally and the dentary anteriorly. The articulars are a small bone contacting the angular anteroventrally and the surangular anterodorsally; it is still articulated with the quadrate in both sides. The surangulars contact the angulars ventrally, the articulars posteriorly and the dentaries anteriorly and constitute the lateral edge of the fossa meckelli. The coronoids are slightly disarticulated and inclined towards the skull, but still contact the surangulars posteriorly and and by its preservational position it can be seen that its anteroventral contact is with the dentary. The dentaries are the largest elements of the lower jaw. They contact the angulars and surangulars posteriorly and the coronoids posterodorsally. The inner side and part of the triturating surface of the left dentary is exposed through the nasal aperture. The dentaries are separated from each other. This is expected since the symphysis is sutured in *A. barretoi* as in *Euraxemys* and *Pelomedusa* (Meylan, 1996), providing a weak point for breakage.

**Hyoid:** Both hyoids are preserved but only the right one can be seen, misplaced toward the posterior portion of the skull. Its anterior portion is located between the axis below and the right lower jaw above. The left hyoid can only be seen through the CT scanning; it is positioned beneath the skull, crushed against the palate. They are laterally flat bones slightly curved near the distal end.

**Carapace:** The carapace length is approximately 200 mm and width is 185 mm (Table 1). Most of the carapace is not visible as the specimen is preserved in ventral view, nevertheless, some peripherals are exposed as well as parts of costals - 1 and 2 next to the front limbs, 4 and 5 next to the hind limbs - which can be seen through the plastral embayment for the limbs. The overall outline of the carapace is ovoid-shaped and anteroposteriorly elongated in contrast with the standard rounded outline of *A. barretoi*. A deep anterior embayment, typical of Araripemydidae, is present. Peripherals 4, 5, 6, 7, 8, 9, 10, 11 from both sides and pygal are exposed in ventral view, except for right peripheral 9 and half of right peripheral 10, which are missing. The pygal and peripherals 11 from both sides are partially covered by some limestone matrix, which could not be removed because it is supporting caudal vertebrae. The carapace ornamentation is mostly unknown – due to the dorsal decubitus position of the specimen – except for the peripherals, which exhibit the fine-pitted texture typical for the Araripemydidae. Peripherals 4, 5, 6 and 7 from both sides are the ones with higher pit density. The neural series can be assessed through CT scanning images along with the inner edges of some costals. The neural series in this specimen is composed of only 8 neurals. Neural bones 2-8 are visible as well as the suprapygal. Neural 2 seems hexagonal and contacts laterally the second costals, posterolaterally the third costals, and posteriorly the third neural. Neural 3 is square-shaped and has only four contacts: the third pair of costals, laterally, and neurals 2 and 4. Neural 4 has a hexagonal shape and contacts anterolaterally the third costals, laterally the fourth costals, and neural bone 3 and 5. Neural 5 has hexagonal shape and contacts anterolaterally the fourth costals, laterally the fifth costals. Neural 8 seems roughly square-shaped - instead of triangular as expected for *A.* *barretoi* – and contacts neural 7 and the eighth pair of costals. It is unclear if it contacts the suprapygal. The shape of contacts of neurals 6, 7 and 8 are not distinguishable. The neural formula is: ? – 6 > 4 < 6 < 6 - ? - ? – 4 (Fig. 5) and it probably were the typical *A. barretoi* formula of: 6 > 6 > 4 < 6 < 6 < 6 > 6 > 3, except for the last neural which appears to have four sides in this specimen. The costals can be assessed as well from the CT scanning. Uniquely, the eighth pair of costals of LP-UFC 722 is relatively thin, being less than half as broad as the seventh pair of costals in the antero-posterior direction.

**Plastron:** The plastron is complete on both sides except for the distal tips from the left epiplastron and entoplastron and small pieces along the middle line. It is smaller than the carapace being 175 mm length and 160 mm wide. The plastron displays both pitted and ridge-and-sulcus ornamentation. A total of nine bones comprise the plastron, which are: a single entoplastron and paired epi-, hyo-, hypo- and xiphiplastra. Mesoplastra are absent. There are two median plastral fontanelles, the anteriormost being much larger than the posteriormost but this must be due the breakage of parts of the hyoplastra and originally there should be three fontanelles. The plastral bridge to the carapace spans for about half the length of the plastron.

The epiplastron is a narrow element with a broad anterior tip and a slender posterior tip. Both epiplastra form an angle less than 45 degrees from the midline. The lateral margin seems slightly curved inward. The epiplastra have extensive medial contact with the hyoplastra and entoplastron but short contact between each other.

The entoplastron has an arrowhead shape with one pointed posteromedial process and two pointed posterolateral processes. Its outer margins contacts the inner margins of the epiplastra by half of its length and the anterior processes of the hyoplastra by the other half. The textural pits from the entoplastron are somewhat bigger than the pits displayed on the other plastral bones, except for the epiplastra which have similar sized pits.

The hyoplastron along with the hypoplastron corresponds for most of the size of the plastron. It has a slender and pointed anterior process that contacts both epi- and entoplastron - only visible on the right hyoplastron as the left is missing. The anterior margin is deeply concave to accommodate the forelimbs (axillar notch). The lateral margin contacts extensively the border of the carapace and is attached to the peripherals 4 and 5. The medial margins of both hyoplastra are broken but they probably composed most of the outline of the two anterior plastral fontanelles. Posteriorly the hyoplastra extensively contacts the hypoplastra along a slightly interdigitating suture. Most of the texture seen on the hyoplastra is a ridge and sulcus type, instead of the rounded pits type seen on the rest on the other plastral bones except hypoplastra, which displays similar ornamentation (Fig. 7).

The hypoplastron has an overall shape similar to the hyoplastron with a deep concavity accommodating the hindlimbs making most of the posterior margin. The rest of the posterior margins contacts the xiphiplastra through a deep interdigitating suture as typical for *A. barretoi*. The anterior margin contacts the hyoplastron as mentioned above. The lateral margin contacts peripheral 6 and 7 of the carapace. The medial edges are not clearly discernible due to the crushing of some vertebrae against the plastron.

The xiphiplastron is a slender element, which has extensive anterior contact with the hypoplastron as mentioned above. Its lateral edges follow the outline of the hypoplastra. The posterior margin is pointed, bending towards the median portion of the plastron to meet its opposite. The medial edges are not discernible, as in the above mentioned other plastral bones, due to preservational conditions. The narrow xiphiplastral tips and the U-shaped anal notch (Fig. 6) indicates that this individual was, probably, a male, following the works of Pritchard (2008) on extant turtles and Cadena (2013) on fossil pleurodires.

**Cervical vertebrae:** The cervical vertebrae are preserved in articulation with each other and both with the skull and the body. Atlas and Axis are both preserved underneath the skull. Although their presence can be asserted through the CT scanning, not much of their morphology could be defined. The remaining cervical vertebrae (3-8) are exposed in ventral view, each joining its neighbour by high angles of approximately 90°.

The centra of the postaxial cervical vertebrae are procoelous. A ventral keel runs along the length of the centra of the postaxial cervical vertebrae, the keel is deeper in cervicals 6 and 7. The keel forms a rounded structure that extends posteriorly beyond the face of the posterior centrum. Subtriangular transverse processes extend laterally on each cervical vertebra. The transverse processes from cervicals 3, 4 and 5 are not developed as the ones from cervicals 6 and 7 – the tips from the transverse process of cervical 7 are remarkably rounded. Cervical 8 is proportionally wider and shorter compared to the remaining vertebrae. As the vertebrae are preserved in ventral view, the zygapophyses are not visible and could not be assessed through the CT scanning.

**Dorsal vertebrae:** The dorsal vertebrae 3-9 along with the first two sacral vertebrae are exposed ventrally through the midline of the plastron due to the ventrodorsal diagenetic flattening of the specimen. Dorsal vertebra 1 lies underneath the entoplastron and the dorsal vertebra 2 is missing except for the anterior tip of its centrum. Dorsal vertebra 3 contacts the ribs from the third and fourth pairs of costals. Dorsal vertebra 4 contacts the ribs of the fourth pair of costals. Dorsal vertebra 6 contacts the rib of the fifth costal. Dorsal vertebra 7 contacts the ribs of the sixth pair of costals. The vertebral centra are hourglass-shaped – remarkably on vertebrae 3 and 4 – and the centrum length decreases from the anterior dorsal vertebrae to the posterior ones, a condition also seen in *T. decorata*. A ventral keel can be seen on dorsal vertebrae 3-6, but they are not as remarkable nor as sharp as the keel from the cervical vertebrae. The remaining dorsal vertebrae are ventrally rounded showing no sign of a keel.

**Caudal vertebrae:** Some caudal vertebrae are preserved although they could not be numbered due the possibility that some of them are lost. The anterior first three are still articulating between each other. Two of them display underdeveloped transversal processes. The remaining ones are scattered and still partially covered by the limestone matrix, which gives support to the elements and thus was not entirely removed during preparation.

**Forelimb:** The left humerus, radius, ulna, carpus and manus are preserved still articulated with each other. The aforementioned bones arise through the plastral opening along with the distal halves of the scapula and coracoid, both still contacting the humerus. The right forelimb has the humerus, the radius, three carpal and three metacarpal bones and three complete phalanges, but only the humerus, the radius, two carpals and the tip of a digit are exposed. All the other bones were only visible through the CT scanning.

The left humerus is preserved fully tridimensional in ventral view. The medial process is broken while the lateral process is entire, a wide intertubercular fossa lies between them. The shaft is almost cylindrical proximally and it flattens and expands distally forming a broad distal end.

The radius is slightly longer than the ulna. Its distal end is narrower than the proximal end which is expanded, while the ulna is almost opposite displaying a slender and cylindrical proximal end and an expanded and wider distal end. Scars for muscle attachment can be seen on the proximal end of the radius as well as on the distal end of the ulna.

The left carpus is preserved in lateral view and the following bones can be assessed: an intermedium fitted in between the distal ends of the radius and ulna, three rounded distal carpals, probably belonging to digits I to III based on their relative positions.

The phalanges of the left manus are compressed and superposed, but still distinguishable. Digits I and II are complete displaying both metacarpals and all phalanges – two for the first digit and three for second. The first metacarpal is about two times shorter and wider than the second one. Digits III and IV are represented only by the last two distal phalanges and digit V only by the tip of the last distal phalanx. Distal phalanges I to III are large and wide elements that probably had claws (exhibiting lateral processes for ungueal sheaths, being arrow-head shaped), whereas distal phalanges IV and V are simple and slender elements that were probably unclawed. The phalangeal formula is 2-3-3-?-?, but it probably displayed the usual 2-3-3-3-3 known to be common among subaquatic turtles.

**Hindlimb:** Both hind limbs are preserved inside the plastral embayment, although disarticulated. The right hind limb presents a femur preserved, in ventral view, almost parallel to the outer edge of the right xiphiplastron; disarticulated tibia and fibula and several pieces of the pes (metatarsals and phalanges). The left hind limb presents an almost fully exposed femur, in lateral view, along with a broken tibia, two metatarsals and two phalanges.

The femur is almost as large as the humerus, but not as stout. About one third of the right femur is underneath other bones, with only the proximal and distal ends exposed. The proximal end shows a wide intertrochanteric fossa, while the distal end shows both ventral and dorsal epicondyle, and an extremely shallow interepicondylar fossa. The left femur is fully exposed displaying a slightly bowed shaft, concave on the ventral side; a rounded head on the distal end, a trochanter major following the curvature of the shaft on the proximal end. The trochanter minor cannot be seen.

The tibia and fibula have similar size, but the tibia is stouter. Both bones are preserved in the right hind limb, although disarticulated. Only the proximal end of the left tibia is preserved (the rest was lost when the slabs with the fossil were cut), while the fibula is missing completely. The shaft of both tibia and fibula are not as bowed as the ones from radius and ulna.

The tarsal elements are scattered, compressed against other limb bones and still partially covered by limestone matrix. Their clear identification is not possible given the misplaced position of the bones, yet some phalanges are clearly distinguishable – two unguals on the right hindlimb and one on the left hindlimb. The unguals are simple, none presenting the arrow-head shape described by Meylan (1996).

***MPSC R 010***

The specimen is a shell only material from the Romualdo Fm., presenting the entire carapace and plastron. MPSC R 010 has about 140 mm in carapace length (Table 1), and displays a light coloration very distinct from the dark brown coloration from the specimens proceeding from Crato Fm.

**Carapace:** The carapace is almost complete – only two peripherals are missing - and is composed of the following bones: a nuchal, 10 neurals, one suprapygal, eight pairs of costals, eleven pairs of peripherals and a pygal. Marks of shell scutes are only observed on pygal and on the peripherals from the left side of the shell. No marks can be seen on the right peripherals except for peripheral 11 nor can be seen on any costals and neurals. A pitted ornamentation can be seen on several costal and neural bones but not on any peripheral bone.

The nuchal is a rather small bone that compose the central portion of the nuchal emargination of the carapace. The anterior edge is circa two times wider than the posterior edge and the lateral margins are inclined giving this bone a triangular shape. It contacts the first pairs of costals laterally, and the first neural posteriorly.

The neural series is comprised of 10 bones (as in the holotype; Price, 1973) instead of the usual nine (e.g. Meylan, 1996). The neural formula is 6 < 6 > 4 < 5 > 6 > 6 > 6 > 6 > 4 < 4 (Fig. 5). It also deviates from the previously reported pattern of *A. barretoi* (Meylan, 1996) for having neural 1 smaller than the second, a fourth neural reaching the left third costal, and besides representing an additional element at the end of series, the last neural (the 10^th^) has four contacts instead of three.

The suprapygal is a quite large bone, circa two times larger than costal 8. It has six contacts, neural 10 and costal pair 8 anteriorly and pygal and peripheral pair 11 posteriorly. The lateral margins of the suprapygal contribute to the last pair of costal fontanelles.

The eight pairs of costals are preserved. The first pair reaches the nuchal emargination precluding contact between the nuchal and the first peripherals. All but the right costals one and two present free rib ends, left costals 2 and 3 have broken rib ends. Fontanelles between the outer edges of costals and the inner edges of the peripherals are visible on both sides of the shell.

Almost all peripherals bones are preserved but for the right peripherals 2 and 3 – which are missing – and right peripherals 1 and 4, which are broken and incomplete. The anterior peripherals are small rectangular bones while the posterior ones are considerably larger and more square-shaped. Peripherals 4, 5, 7, 8, 9 and 10 from the left side of the shell and peripherals 11 from both sides present marginal scutes marks.

The pygal is a single square-shaped bone quite similar the posterior peripherals. It has about the same size of peripheral 11 but is a little narrower. A groove of the last pair of marginal scutes and the last vertebral scute is clearly visible.

**Plastron:** The plastron is complete with all bones still tightly sutured to each other. It has around 100 mm in both length and width (Table 1). The plastral bones do not show morphological variation and follow the same pattern described for LP-UFC 722. A densely pitted ornamentation is widespread on the surface of every plastral bone, but there are no signs of the ridge-and-sulcus ornamentation. Remains of synthetic resin used in the adulteration of the specimen are scattered through the plastron.

The right epiplastron distal tip is broken as well as the posteromedial process of the entoplastron and the inner edges of the hyoplastra. Three fontanelles are present. The first surrounded by the entoplastron anteriorly, and by the pair of hyoplastra posterolaterally. The second is surrounded by both pairs of hyoplastra and hypoplastra, and the third one is surrounded by both pairs of hypoplastra and xiphiplastra. The xiphiplastra exhibits rounded distal tips and a V-shaped anal notch, being here interpreted as a female.

***MPSC R 2107***

The specimen is an incomplete plastron and it is preserved exposed in ventral view on a single limestone slab. MPSC R 2107 has about 150 mm in both length and width (Table 1) and is composed of the following paired bones: hyoplastra, hypoplastra and xiphiplastra, presenting three fontanelles. The pair of epiplastra and the single entoplastron are missing (Fig. S1). The hyoplastra are well preserved, showing no signs of breakage, even though the pair is slightly disconnected through the strongly interdigitated medial suture. The anterior edges of the hyoplastra presents an outline that indicates that the missing entoplastron had the typical arrowhead shape of *A. barretoi*; it also presents most of the outline of the anteriormost fontanelle. The posterior edges contacts the hypoplastra, and compose about a half of the outline of the second fontanelle. The hypoplastra are complete – except for the posterior portion of the outer edge of the left hypoplastron - still contacting both pairs of hyoplastra and xiphiplastra, but not contacting each other at the midline. The anterior edges have a large contact with the hyoplastra, and compose most of the outline of the second plastral fontanelle. The specimen shows the pectoral, abdominal and femoral scutes pattern like other specimens of *A.* *barretoi* (see Meylan, 1996). The posterior edges contact the xiphiplastra through a strongly interdigitated suture – a condition known to *A. barretoi* – and compose great deal of the third plastral fontanelle. The hypoplastra displays marks of the femoral scutes. Both xiphiplastra are preserved, except the distal end of the left xiphiplastron, which is broken. The xiphiplastra presents marks of the anal scutes, and have a large contact with the hypoplastra anteriorly, being still connected to each other. The anteromedial edges compose the outline of the third fontanelle, along with the hypoplastra. The distal tip of the right xiphiplastron – which is entirely preserved - is narrow and elongated, and its anal notch is U-shaped in the same way observed in the xiphiplastra from the LP-UFC 722, being thus also interpreted here as a male. All plastral elements present a finely grooved ornamentation, lacking any signs of pitting. The middle plastral fontanelle is ovoid in shape, wider than long. The last plastral fontanelle is polygonal, apparently pentagonal, longer than wide with its length subequal to the width of the second fontanelle.

***UFRPE 5302***

The specimen is the posterior portion of the carapace and it is preserved on a single limestone slab, proceeding from the Crato Fm. The material is about 80 mm in length, and 130 mm in maximum width (Table 1).

Peripherals 9, 10 and 11 are preserved on both sides. A small fragment of peripheral 8 is still preserved on the right side, while on the left side the complete peripherals 5, 6, 7 and 8 are preserved, as well as most of peripheral 4. The pygal bone has a triangular shape instead of the usual square shape. It does not contact the suprapygal because peripherals 11 from both sides meet at the midline, thus precluding the contact between pygal and suprapygal. Costals 6, 7 and 8 are preserved on both sides - costal 5 is complete on the left side, as well as a fragment of costal 4 (Fig. S1). Costals 8 meet at the midline, a common condition in both living and fossil turtles although not universal.

The neural series is incompletely preserved, displaying the last three neurals (Fig. 5). In every specimen herein analyzed, neural 9 (as well as neural 10, if ever present) locates itself between the eight pair of costals. Since there is no neural between pair of costals 8 in UFRPE 5302, it seems likely that neurals 9 and 10 are lacking, leaving it a total of eight neurals only. Such a condition would be similar to what is observed in *T. decorata*: the neural series comprises only eight neurals, and the pair of costals 8 meet completely in their midlines, without any neural in between them, where neurals 9 and 10 would be present in most specimens of *A. barretoi* (Sereno & ElShafie, 2013).

Costals, neurals and suprapygal exhibit a fine pitted ornamentation while the peripherals exhibit the ridge-and-sulcus texture instead. Groove marks of shell scutes - the last three vertebral and the last two pairs of lateral along with several marginals - can be clearly observed on this specimen.

***MN 6949-V***

This Romualdo Fm. specimen includes a fairly complete skull, mandible, hyoids, almost complete shell (lacking only the anterior region of the epiplastra), two cervical vertebrae, and a complete manus, all in exquisite three-dimensional preservation. Some other appendicular elements are present, though obscured by matrix. It has been briefly mentioned before: the manus was figured by Oliveira & Kellner (2007a) and the cervical vertebrae were described by Mariani & Romano (2017). The shell and skull are described here for the first time.

**Skull:** The skull is almost complete and most sutures between the elements are well distinguishable. The rostral region is intact, presenting an elongate aspect with a round anterior margin. The premaxillae and maxillae exhibit several neurovascular foramina, similarly to the dentary underneath. The premaxillae protrude relative to the dentary, similarly to THUg 1907 (Gaffney *et al.*, 2006); and their ventral surface are obscured by a small layer of matrix.

The prefrontals are complete and exhibit a round anterior margin, contacting the frontals posteriorly and the maxillae laterally. The orbits are ovoid in shape and bounded by the prefrontals, maxillae, frontals, postorbitals and jugals. They are close to each other and dorsally located on the skull.

The frontals begin anteriorly close to the anterior margin of the orbit, contacting the prefrontals anteriorly, the parietals posteriorly and the postorbital posterolaterally. These bones exhibit a somewhat straight suture between them.

Posteriorly, the parietals are trapezoidal in shape and contacted anteriorly by the frontals and postorbitals. They are the largest bones in the skull roof. The sutures between the two parietals, as well as between each parietal and the frontals, are interdigitated, but not strongly.

The postorbital contacts the jugal anterolaterally, quadratojugal posterolaterally, the frontal anteromedially and the parietal posteromedially. (Gaffney *et al.*, 2006).

The jugal contacts the maxilla anteriorly with a linear suture, and the suture with the maxilla is unclear. Similarly to the maxillae, premaxillae and dentary, the anterior region of this bone is covered by small neurovascular foramina.

The left quadratojugal is almost completely lost, except for the anterior region contacting the jugal. The right one is almost complete and reveals an anterior contact with the jugal and a posterior one with the quadrate and squamosal.

In dorsal view, the quadrate contacts the opisthotic posteromedially, the prootic anteromedially and, apparently, the squamosal posteriorly. The foramen stapediotemporale is surrounded by the quadrate and prootic bones (Fig. 3). Posteriorly, another large foramen pierces the quadrate, partially bounded medially by the opisthotic and posteriorly by the squamosal as well (Fig. 3), similarly to AMNH 24454 and THUg 1357 (Gaffney *et al.*, 2006), but unlike AMNH 24453 (Meylan, 1996) and LP-UFC 722. In ventral view, close to the contact region with the prootic, this bone exhibits a very small foramen (Fig. 3), similarly to AMNH 24454 (Gaffney *et al.*, 2006). The right stapes is exposed, not enclosed, and can be seen in what seems to be a natural contact with the quadrate, corresponding to the same region where the incisura columellae auris of AMNH 24454 can be seen (Gaffney *et al.*, 2006).

The palatines contact the maxillae distally and the pterygoids posteriorly. Contact with the jugal cannot be seen, as the distal margins are partially obscured by matrix and the mandible.

The pterygoids are paired bones that meet medially anterior to the tip of the basisphenoid in AMNH 24454 and THUg 1537 (Gaffney *et al.*, 2006). This medial contact between the two bones cannot be assessed in MN 6949-V due to a breakage in this region.

The basisphenoid is a single large bone, trapezoidal in shape, anterolaterally bounded by the two pterygoids, in contrast with *T. decorata*, in which the basisphenoid reaches the palatine (Sereno & ElShafie, 2013 Fig.14.7a,b), and posteriorly by the prootics and the basioccipital.

The prootic is a paired bone that, in dorsal view, contacts the parietal, quadrate, opisthotic, pterygoid and supraoccipital. It encloses the foramen stapediotemporal, which is also slightly bounded by the quadrate. In ventral view, the prootic contacts the basisphenoid, quadrate, opisthotic, pterygoid and supraoccipital. It encloses entirely the foramen posterius canalis carotici interni.

The opisthotic contacts the prootic anteriorly, the quadrate anterolaterally, the exoccipitals and the supraoccipital medially and the squamosal laterally. Both opisthotics have lost their distal ends, the right one in a greater extent than the left one. Its medial margin, though incomplete, is clearly concave, the same condition seen in AMNH 24454 (Gaffney *et al.*, 2006) and AMNH 24453 (Meylan, 1996), and unlike THUg 1357 (Gaffney *et al.*, 2006) and LP-UFC 722, which show a convex medial margin. In ventral view, the foramen jugulare posterius can be seen in a closed condition, bounded mostly by the exoccipital but also by the opisthotic anteriorly.

The squamosal can be seen contacting the quadrate, the opisthotic and the quadratojugal through a very narrow suture.

The exoccipital contacts the opisthotic laterally in both dorsal and ventral views, the basioccipital anteriorly and medially in ventral view, and the supraoccipital anteriorly in dorsal view. Two hypoglossal foramina (XII) pierce the exoccipital, in the ventral region of the bone, more medially than the foramen jugulare posterius. These two hypoglossal foramina are located one dorsal to the other, instead of one dorsolateral to the other as seen in AMNH 24454, THUg 1907, THUg 1357 (Gaffney *et al.*, 2006) and *T. decorata* (Sereno & ElShafie, 2013). There are no signs of the exoccipital-opisthotic foramen (“extra” foramen) seen in AMNH 24454 (Gaffney *et al.*, 2006).

The basioccipital contacts the exoccipitals laterally and the basisphenoid anteriorly. The basioccipital forms the ventral region of the occipital condyle (about a third of the structure), and the exoccipitals take part on the laterodorsal regions of the structure. The foramen magnum is more or less round, similar to AMNH 24454 and unlike THUg 1357 in which it is rather triangular (Gaffney *et al.*, 2006).

**Mandible:** The mandible is preserved in natural occlusion with the skull, preventing observation of its dorsal and medial aspects. It is completely preserved. In lateral view, the sutures delimitating each bone can be easily recognized and are the same as in AMNH 24454 and THUg 1907 (Gaffney *et al.*, 2006).

The dentaries are paired bones that meet medially*.* They are sutured and finely pierced by small neurovascular foramina on the anterior region, reflecting the condition seen in the upper jaw (in the premaxillae, maxillae and jugals). It contacts the coronoid posterodorsally, the surangular posteriorly and the angular posteroventrally.

The coronoid is a small paired bone that forms the coronoid process together with the dentary in lateral view. A case of asymmetry can be seen regarding this bone. In MN 6949-V, the right coronoid is quite small and forms the posterior half of the coronoid process. The anterior half is formed almost entirely by the dentary, with only a slender dorsal projection of the coronoid, similarly to what is seen in AMNH 24454 and THUg 1907 (Gaffney *et al.*, 2006). The left coronoid, on the other hand, extends onto and occupies entirely the anterior half of the process. Again, the same morphology is seen in AMNH 24454, and in THUg 1907 the coronoid occupies most of the anterior half of the coronoid process (Gaffney *et al.*, 2006).

The surangular contacts the dentary anteriorly and ventrally, the coronoid anterodorsally, the angular posteroventrally and the articular posteriorly. Its posterior region is pierced by a small foramen nervi auriculotemporalis, similar to AMNH 24454 and THUg 1907 (Gaffney *et al.*, 2006).

The articular constitutes the posterior region of the mandible, forming the articular surface for the quadrate. It exhibits a round posterior margin on both sides.

**Carapace:** The carapace comprises a nuchal, 10 neurals, a suprapygal, eight pairs of costals, 11 pairs of peripherals and a pygal (Fig. 4). It is 165 mm in length and 140 in maximum width (Table 1), exhibiting the squared outline already reported in the literature (Price, 1973; Meylan, 1996). All shell elements are densely covered by the typical pits (as described by Meylan, 1996), without any presence of ridge-and-sulcus ornamentation. The pits are particularly conspicuous in the costals, neurals and suprapygal, being fainter in the peripherals. The last two pairs of costals do not form costal fontanelles. Neurals 1-3 and 5-7 are hexagonal, neurals 4, 8 and 9 are squared, and neural 10 is triangular. The neural series is: 6 = 6 < 4 < 5 > 6 > 6 > 6 > 6 > 4 > 4. Neural 1 contacts the nuchal, neural 2 and pairs of costals 1 and 2; neural 2 contacts neurals 1 and 3 and pairs of costals 2 and 3; neural 3 contacts neurals 2 and 4 and pair of costals 3 only; neural 4 contacts pairs of costals 4, right costal 3 and neurals 3 and 5; neural 5 contacts pairs of costals 4 and 5 and neurals 4 and 6; neural 6 contacts pairs of costals 5 and 6 and neurals 5 and 7; neural 7 contacts pairs of costals 6 and 7 and neurals 6 and 8; neural 8 contacts pairs of costals 7 and 8 and neurals 7 and 9; neural 9 contacts pair of costals 8 and neurals 8 and 10; neural 10 contacts neural 9, pair of costals 8 and the suprapygal. The observable marginal scutes are similar to those already described by Meylan (1996) except for the last vertebral scute which is wider than long in MN 6949-V, similar to *T. decorata* (Sereno & ElShafie, 2013) and unlike AMNH 23343 (Meylan, 1996).

**Plastron:** The plastron exhibits a cruciform morphology no different from previously described conditions (Price, 1973; Schleich, 1990; Meylan, 1996), composed of an entoplastron and paired epiplastra, hyoplastra, hypoplastra and xiphiplastra. The preserved remains totalize 132 mm in length and 136 mm in width (Table 1); though the original length cannot be assessed due to the incompleteness of the epiplastra. The suture between the hyoplastra is particularly well-interdigitated. The xiphiplastra exhibit a U-shaped anal notch. All plastral elements are conspicuously pitted, without any ridge-and-sulcus ornamentation (as shown on left hyo- and hypoplastron, Fig 12). The only regions with smooth surfaces are the margins of the axillary notch (in the epiplastra and hyoplastra) and the distal margin of the inguinal notch in the hypoplastra. There are three well-developed fontanelles, an anterior “heart-shaped” one, an oval medial one (wider than long) and a longer than wide posterior one of unclear shape due to matrix obliteration. Its length is slightly shorter than the width of the middle fontanelle (Fig. 4).

**Manus:** The manus of MN 6949-V was figured by Oliveira & Kellner (2007) who briefly described the phalanges: they are elongate and blunt, not arrow-shaped (Oliveira & Kellner, 2007a; Fig. 4).

***MN 6743-V***

This specimen is composed of a partial shell with an almost complete carapace and fragmentary plastron (Fig. S3).

**Carapace:** The anteriormost region of the carapace is missing, with the anterior region of the pair of costals 1 being lost, alongside with the nuchal. Posteriorly, nine neurals are present. The neural series is: ? – 6 < 4 > 6 < 6 = 6 > 6 > 4 > 3, existing a contact between neural 9 and the suprapygal.

**Plastron:** The plastron exhibits fragments of both hyoplastra, both hypoplastra, and of the right xiphiplastron. All these elements are ostensibly covered by a fine ridge-and-sulcus ornamentation. All three fontanelles are present, but their exact shapes cannot be assessed.

***MN 6744-V***

This specimen comprises an almost complete shell with two cervical vertebrae and some indeterminate appendicular elements partially obscured by matrix. The carapace is 150 mm in both length and width (Table 1). The peripherals were lost, but their impressions reveal the typical pitted ornamentation. Neurals and costals also exhibit pitted surfaces. Small costal fontanelles are present (Fig. S2).

The neural series is: 6 > 6 < 4 > 6 > 6 > 6 > 6 > 4 > 3. Neural 9 is odd in presenting a round posterior margin and contacting only neural 8 and pair of costals 8, which meet medially and prevent contact between neural 9 and the suprapygal (Fig. 5). Interestingly, this is similar to the specimen BSP 1981 I 38 which also presents a rounded neural 9 that does not contact the suprapygal, with pair of costals 8 meeting medially (Schleich, 1990), but not entirely as in UFRPE 5302 or *T. decorata* (see Sereno & ElShafie, 2013).

***DGM 346-LE***

This specimen consists of an almost complete shell from the Romualdo Fm. The plastron is complete and well-preserved, but the carapace lacks most of its dorsal bone surface.

**Carapace:** The carapace is 210 mm in length and 167 mm in width (Table 1). The neural series is almost completely lost and, in fact, the dorsal vertebrae are visible in dorsal view. Despite this, it is clear from the preserved remains that there are no costal fontanelles at least on the last two pairs of costals (Fig. S2), similarly to MN 6949-V. The few preserved bone surfaces present the typical pitted ornamentation.

**Plastron:** The plastron is complete, being 173 mm in length and 162 mm in width (Table 1). All plastral elements are richly pitted, though not as conspicuously as in MN 6949-V (as shown on left hyo- and hypoplastra, Fig. 7). The hyoplastra, epiplastra and hypoplastra also present some smooth margins surrounding the axillar and inguinal notches, similarly to MN 6949-V. The sutures between each hyoplastra and hypoplastra are strongly interdigitated. The three typical plastral fontanelles are present, individualized, exhibiting perfectly preserved margins. The anterior one is heart-shaped and slightly asymmetrical, being the right side larger than the left side. The middle one is ovoid in shape, wider than long as with other known specimens (e.g., Meylan, 1996), and not polygonal as in the holotype. The posterior one is interesting in being polygonal instead of ovoid, being approximately pentagonal. It longer than wide, its length subequal to the width of the middle fontanelle. The last fontanelle of the holotypic specimen is polygonal as well, though comparatively much smaller than the middle fontanelle. The anal notch is U-shaped, conforming with a male (Fig. S2).

***DGM 1449-R***

This specimen is a shell from the Romualdo Fm. with only the carapace visible. Remains of both humeri and cervical vertebrae 8 and 7 can also be seen, but are too damaged for any detailed description. It was included here due to the morphology of the costal fontanelles, and for being the second largest specimen we had access to, with a carapace length of 223 mm and width of 225 mm (Table 1).

Its surface is much damaged and not much can be observed. Preserved surfaces of costals exhibit a fine pitted ornamentation. The peripherals exhibit pits as well as several ridges and sulcus.

The neural series is badly preserved. Unfortunately, the neural formula cannot be completely assessed. Above the suprapygal, it can be seen that the last pair of costals contact at their midlines, without any trace of a neural between them, and thus a lacking contact between the last neural and the suprapygal. This lack of any neurals between the pair of costals 8 probably means the specimen, most likely, only had 8 neurals, similarly to *T. decorata* (Sereno & ElShafie, 2013).

There are carapacial fontanelles through costals 2 to 8, and even between the costal 8 and the suprapygal. Only the pair of costals 1 does not form fontanelles. However, all fontanelles are relatively diminutive (Fig. S4).

***MPSC R 2308***

This specimen is a shell only material coming from the Romualdo Fm. The specimen is partially preserved presenting a carapace (190 mm in length and 173 mm in width; Table 1) and plastron only.

**Caparace:** Most of the carapace bones are still covered by matrix but some of the costals are visible, mainly on the right side of the specimen, as well as some peripherals and the pygal. Impressions of the last three right ribs can be seen. All costals and some peripherals exhibit a dense pitted ornamentation (Fig. S2).

**Plastron:** Most of the plastral bones are still covered by matrix, like the carapace. The left posterior process of the entoplastron is exposed along with portions of the hyoplastra, hypoplastra and xiphiplastra (Fig. S2). Both hyoplastra and hypoplastra exhibit a densely pitted ornamentation on their ventral surfaces. In part of the right hyoplastra the bone surface has been lost, with a striated inner bony layer exposed. Three fontanelles are visible. The shape of the first fontanelle cannot be assessed due to the incompleteness of bones surrounding it. The middle fontanelle seems to be polygonal, similar to the holotype, whereas the last fontanelle is oval, the longest axis being the antero-posterior one. The xiphiplastra exhibit narrow tips and a U-shaped anal notch indicating that this individual was, possibly, a male.

***MPSC R 874***

This material comes from the Romualdo Fm. and presents an almost complete carapace, except for a small breakage on the posterior region and a badly preserved plastron, of which only the overall outline and two fontanelles can be seen, of which the shape cannot be assessed. No sutures nor the exact outline of the xiphiplastra can be observed. The following carapace bones can be seen: nuchal, suprapygal, pygal, all eight pairs of costals, most peripherals and 10 neurals. A pitted ornamentation is observed on several costals, neurals and peripherals, mainly on the posterior region of the carapace. No ornamentation can be seen on the plastron due to its poor preservation.

***MPSC R 134***

This specimen is a shell only material coming from the Romualdo Fm. presenting light colored part and counterpart of the carapace. MPSC R 134 is the largest individual seen on this study, having circa 250 mm in lenght and width (Table 1), and displaying a very rounded carapace outline. All bones seem to be preserved, although the anteriormost peripherals are hard to distinguish. This individual exhibits 10 neurals. The peripherals – in special the posteriormost ones – are proportionally larger than in other *A. barretoi* individuals. A dense pitted ornamentation can be observed on the costals, neurals and peripherals. The intercostal fontanelles are very small (Fig. S4), presumably due to the individual late ontogenetic stage.

***MPSC R 137***

MPSC R 137 comes from Romualdo Fm. and has only the carapace preserved, with most of the bones displaying a dark brown coloration more similar to the Crato Fm. material than to Romualdo Fm. MPSC R 137 is a small individual having 140 mm in lenght and 150 mm in width (Table 1). Almost all bones are preserved except for the right costals which were lost, but are represented by the internal impression alongside the internal impressions of the associated ribs. A pitted ornamentation is observed on the neurals and costals but not on the peripherals. MPSC R 137 presents very large intercostal fontanelles (Fig. S4), presumably due to the early ontogenetic stage of the individual.

***SMNK PAL*** *(no number)*

We report here a new specimen deposited in the collection of SMNK PAL, still lacking a number and final preparation. This specimen is preserved in a calcareous nodule from the Romualdo Formation. The carapace is almost complete, lacking small fragments of left costals 1 – 4, right costals 5 – 7. The pygal and peripherals 9 – 11 on both sides are almost completely lost, as well as 7 – 8 on the right side. The plastron is presumably enclosed within the nodule. It also includes an almost complete cervical series and a skull, only partially exposed in right view pending further preparation. Future preparation could provide information concerning key features, such as the opisthotic and the foramen jugulare posterius.

The carapace is well-preserved and almost complete. It presents a nuchal, 10 neurals, one suprapygal, a pygal, 11 pairs of peripherals and eight pairs of costals. The neural series is as follows: 6 < 6 > 4 = 6 > 6 > 6 > 6 > 6 > 4 > 3. The suprapygal contacts the last neural. All carapacial elements exhibit a finely pitted ornamentation. The carapace is oval in shape (Fig. S4).

**Revisited Specimens**

***DGM 756-R***

This is the holotypic specimen, originally described by Price (1973). Ever since its original description, this material has awaited to be figured in high-quality.
 The specimen comprises almost complete carapace and plastron, lacking only part of the anterior border. On the left side, only peripherals 1 and 2 are completely missing, alongside part of costal 1 and peripheral 3. On the left side, costal 1 is lacking almost entirely, as well as the peripherals 1 through 4 and part of peripheral 5. The nuchal is almost completely lost as well. Both femora are present as well, a complete right one and a partial left one. Their embedment in matrix prevents the report of any details.

**Carapace:** The carapace is composed of eight pairs of costals, probably 11 pairs of peripherals, a nuchal, 10 neurals, a suprapygal and a pygal. The neural series is as follows: 6 < 6 > 4 = 6 > 6 > 6 > 6 > 6 > 4 > 3. The last neural contacts the suprapygal. The external surface of the carapace is almost completely gone, except for part of left costals 2 through 5 and peripherals 6 and 7, where a finely pitted ornamentation can be seen. Very diminutive carapacial fontanelles can be seen between costals 4 through 6 and the peripherals (Fig. S2).

**Plastron:** The plastron is cruciform in shape. The specimen lacks its epiplastra and entoplastra entirely, together with parts of both hyoplastra. The hypoplastra and xiphiplastra are complete (Fig. S2). The mesoplastra are absent, as in every other *A. barretoi* specimen with a visible plastron. As with the carapace, the plastron is covered in a pitted ornamentation. There is no sulcus-and-ridge pattern in any plastral region. The two posterior fontanelles can be seen. The middle one is diamond-shaped, wider than long. The posteriormost one is diamond-shaped as well, longer than wide, and smaller than the middle one. The anal-notch is U-shaped, though the xiphiplastral extremities are not as prominent as in LP-UFC 722 or MN 6949-V.

***MN 6637-V***

This specimen comprises several elements, including a complete plastron, dorsal vertebrae, scapulae, coracoids, manual phalanges, pelvis, femurs and a humerus fragment (Fig. S3), other than the as-yet undescribed internal mold of the carapace. Most of the specimen has been described in detail by Kischlat & Campos (1990). We provide here some further details concerning the carapace, the plastron and the phalanges, together with better-resolution photos of other postcranial elements already described by the previous authors.

**Carapace:** In this specimen, only an internal impression of the carapace had been preserved on the matrix (Fig. S3). This positive impression has been reproduced in a negative resin mold prior to chemical preparation, which removed the matrix entirely. There can be seen eight pairs of costals. Other carapacial elements are not clearly visible. Distal fragments of costals 2 through 4 can be seen in dorsal view attached to the plastron. They exhibit a pitted ornamentation.

**Plastron:** The ventral face of the plastron has been described before (Kischlat & Campos, 1990), with the visceral face having been still embedded in matrix. The material went through acid preparation under transfer. Now, the ventral surface of the plastron is embedded in resin and the visceral surface is exposed. All plastral elements are preserved. The plastron exhibits the typical cruciform shape, similar to other specimens. The first plastral fontanelle is heart-shaped. The middle fontanelle seems to have been oval, wider than long. The shape and size of the posterior fontanelle is unclear due to the presence of matrix. The resin is rather opaque and details of the ventral surface of the plastron, such as ornamentation, can no longer be assessed. The anal-notch is U-shaped (Fig. S3).

**Phalanges**: The phalanges are reported to come from the right manus, as could have been observed prior to preparation (Kischlat & Campos, 1990). These phalanges are here tentatively identified as belonging to the third manual digit, based on comparison to the proportions between the phalanges and between their length/width (see Table 2) according to the description by Meylan (1996). The ungual lacks only its tip. It is arrow-head shaped, exhibiting well-developed lateral processes for ungual sheath attachment.

***SMNK PAL 3979***

This specimen is the holotype of the proposed species *A.* “*arturi*”. It is composed of a partial shell and partial left hindlimb (tibia, fibula and pes), preserved in ventral view in a limestone plate from the Crato Formation. It also includes a dorsal vertebra and a tiny portion of the left xiphiplastron.

**Carapace:** The incomplete carapace exhibits a partial peripheral series and costal series, exposed in ventral view and partially obscured by the plastron. There are present peripherals 2-9 and costals 1-7. The maximum length of the fragment is 180 mm, and the complete carapace must have been slightly longer than that. It is oval in shape, what had been considered diagnostic for *A.* “*arturi*”. Oval carapaces are equally seen in LP-UFC 722, DGM 346-LE, MN 6744-V, and MPSC R 134 and BSP 1981 I 38. The peripherals exhibit a pitted ornamentation.

**Plastron:** Only lateral portions of the left hyo- and hypoplatron are present, exposed in ventral view. There are relatively slender bones, indicative of the typically cruciform plastron seen in *A. Barretoi*. The bone surface has been lost in some regions, particularly in most of the hypoplastron. Where the bone surface is well preserved (most of the hyoplastron and a small region of the hypoplastron), a finely pitted ornamentation can be seen. No signs of ridge-and-sulcus ornamention are present. In the hyoplastron, where the ventral surface of the bone has been lost, the exposed inner layer of bone is striated (not to be confused with a superficial ridge-and-sulcus ornamentation).

**Unguals:** As described by Fielding, Martill & Naish (2005), the unguals of SMNK PAL 3979 are simple. They are relatively slender, matching the morphology seen in MN 6949-V. They lack any signs of lateral processes for ungual sheath attachment (Fig. S7), oppositely to LP-UFC 722, MN 6637-V, AMNH 24453 and AMNH 24454.

***BSP 1977 I 1***

This specimen, together with BSP 1981 I 38, was originally described by Schleich (1990). This specimen consists of an almost complete shell, lacking only small portions of some elements, as well as some limb elements including a manual ungula (Fig. S3). Other elements (cervical vertebrae, right humerus, radius, ulna, carpal and metacarpal elements; both tibiae, fibulae, tarsals and metatarsals) are also preserved, but still partially embedded by matrix, preventing detailed descriptions. Here, we provide some details concerning the neural series, ornamentation and ungual condition.

**Carapace:** The carapace is oval in shape. The neural series is complete, composed of ten neurals, as described by Schleich (1990). The neural formula is as follows: 6 < 6 > 4 < 6 > 6 = 6 > 6 > 4 > 3. The surfaces of the costals are almost completely lost. Where preserved, they exhibit a finely pitted ornamentation, as well as on the nuchal, neurals and peripherals. Small carapacial fontanelles are present.

**Plastron:** All elements are preserved. The bone surface is well-preserved on most regions, but not all. Where it is present, a finely pitted ornamentation can be seen. Where the surface has been lost, the inner bony layer is exposed, showing a striated surface, similar to SMNK PAL 3979. Three plastral fontanelles are present, but their margins have been damaged and their original sizes and shapes cannot be assessed. Interdigitations in the sutures between hyo- and hypoplastra and hypo- and xiphiplastra are small (Fig. S3).

**Manus:** Of the right limb, there is the first metacarpal and the first digit. The ungual is simple, lacking any lateral process for ungual sheath attachment.

***BSP 1981 I 38***

**Carapace:** The carapace is squared in shape. It is almost complete, with nuchal, nine neurals, pygal, suprapygal, eight pairs of costals, fragmentary peripherals 1 – 6 and almost complete peripherals 7 – 11 on the left side, fragmentary peripherals 1 – 9 on the right side and complete right peripherals 10 – 11 (Fig. S3). The neural series is complete, with the following formula: 6 < 6 > 5 < 6 > 6 > 6 > 6 > 6 > 3 (Fig. 5). There is no contact between the last neural and the suprapygal. The bony surface is well-preserved in most costals and neurals, which exhibit a finely pitted ornamentation. The bony surface of the peripherals, along with the ornamentation pattern, have been lost to over-etching by acid preparation according to Schleich (1990).

**Plastron:** The plastron lacks only the entoplastron and epiplastra. The bony surfaces of both xiphiplastra were completely lost due to over acid preparation, as stated by Schleich (1990), presenting a smooth surface which lost the original superficial ornamentation. The xiphiplastra exhibits a V-shaped anal notch characteristic of females (Fig. 6), following the works of Pritchard (2008) and Cadena (2013). Where present, the surface of the hyo- and hypoplastra show both pits and ridge-and-sulcus patterns, though some areas (anterior regions of the hyoplastra and lateral regions of hyo- and hypoplastra) have also been smoothened by the acid preparation. The anterior fontanelle exhibits a damaged anterior margin, preventing assessement of its shape. The middle fontanelle is oval, wider than long, larger than the posterior fontanelle. The posterior fontanelle seems to have been triangular (Fig. S3).
