# Supplementary figures and images for: Interpreting character variation in turtles: Araripemys barretoi (Pleurodira: Pelomedusoides) from the Araripe Basin, Early Cretaceous of Northeastern Brazil
Source: PeerJ. 2020 Sep 29;8:e9840. doi: 10.7717/peerj.9840 (PMC7531347; doi:10.7717/peerj.9840)

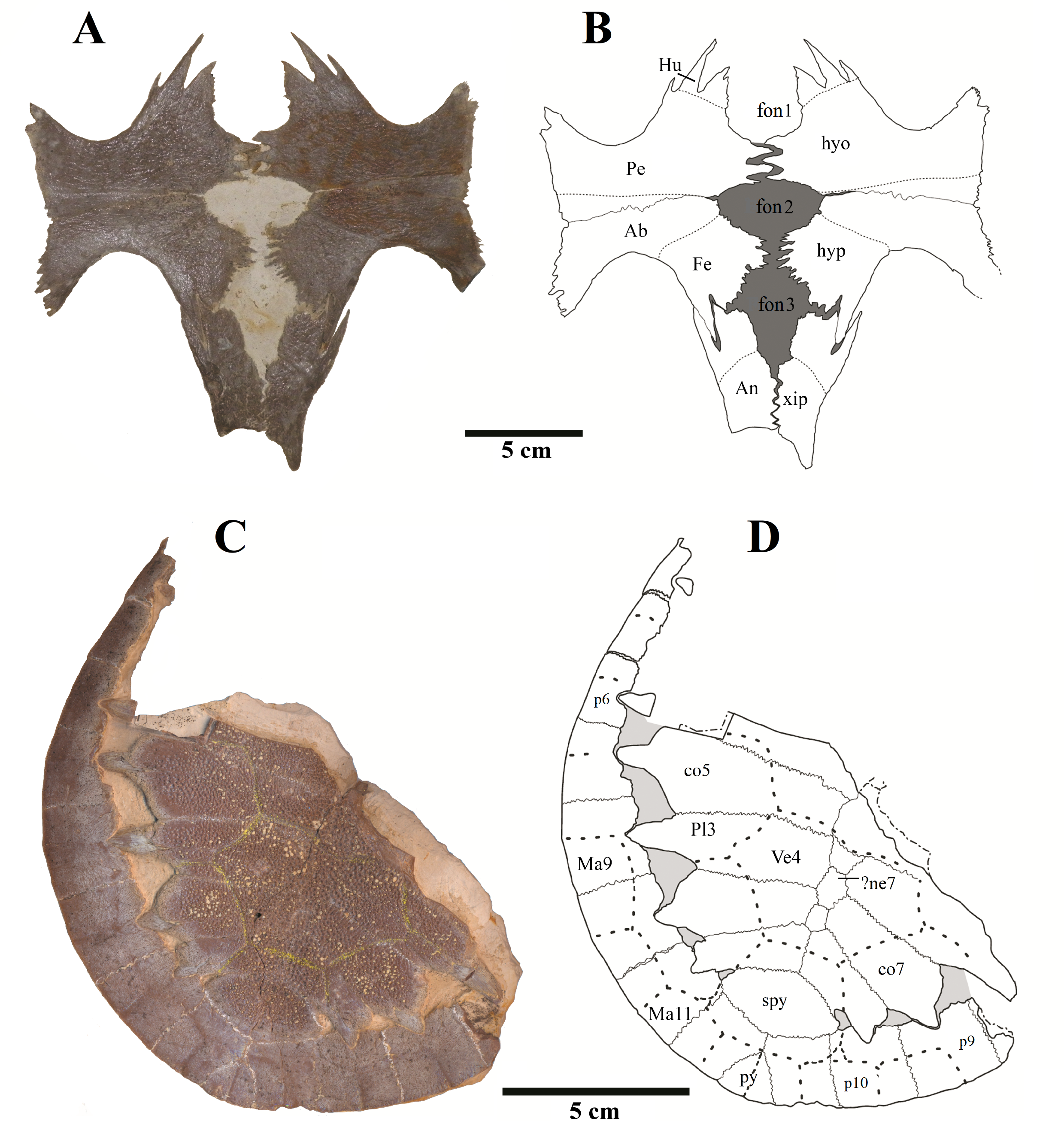

Supplement: Supplemental Information 3 — A) MPSC R 2107, plastron in ventral view and B) schematic drawing. Photo and drawing by Thales Nascimento. C) UFRPE 5302, carapace in dorsal view and D) schematic drawing. Photo and drawing by Thales Nascimento. Abbreviations: Ab – abdominal scute, An – anal scute, co – costal, Fe – femoral scute, Fen – fenestra, Hu – humeral scutes, hyo – hyoplastron, hypo – hypoplastron, Ma – marginal, ne – neural, Pe – peripherals, Pl – pleural scute, Py – pygal, Spy – suprapygal, Ve – vertebral scute, Xip – xiphiplastron. [file peerj-08-9840-s003.png]

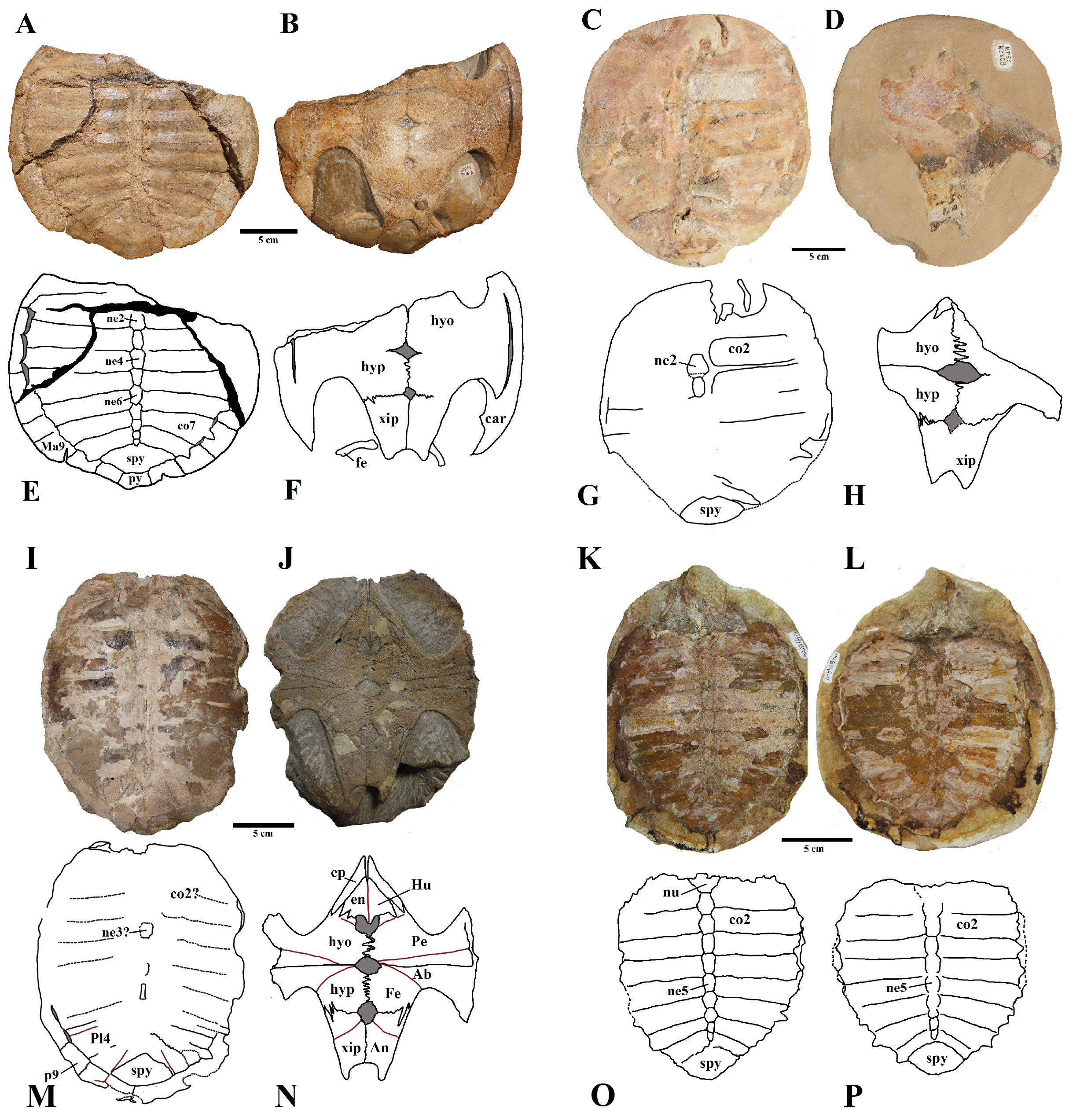

Supplement: Supplemental Information 4 — Holotype (DGM 756-R), A) carapace, dorsal view and B) plastron, ventral view. Photo by Rodrigo V. Pêgas. MPSC R 2308, C) carapace in dorsal view and D) plastron in ventral view. Photo by Renan Bantim. DGM 346-LE. E), F), G), and H), respective schematic drawings. I) Carapace in dorsal view and J) plastron in ventral view. Photo by Rodrigo V. Pêgas and Thales Nascimento. MN 6744-V, K) part with carapace in dorsal view and L) counterpart with carapace remains in internal view. M), N), O) and P), respective schematic drawings. Photo by Rodrigo V. Pêgas and Thales Nascimento. Abbreviations: car – carapace, co – costal, en – entoplastron, ep – epiplastron, fe – femur, Hu – humeral scute, hyo – hyoplastron, hyp – hypoplastron, Ma – marginal, n – neural, nu – nuchal, pe – peripheral, Pl – pleural, py – pygal, spy – suprapygal, xip – xhiphiplastron. [file peerj-08-9840-s004.png]

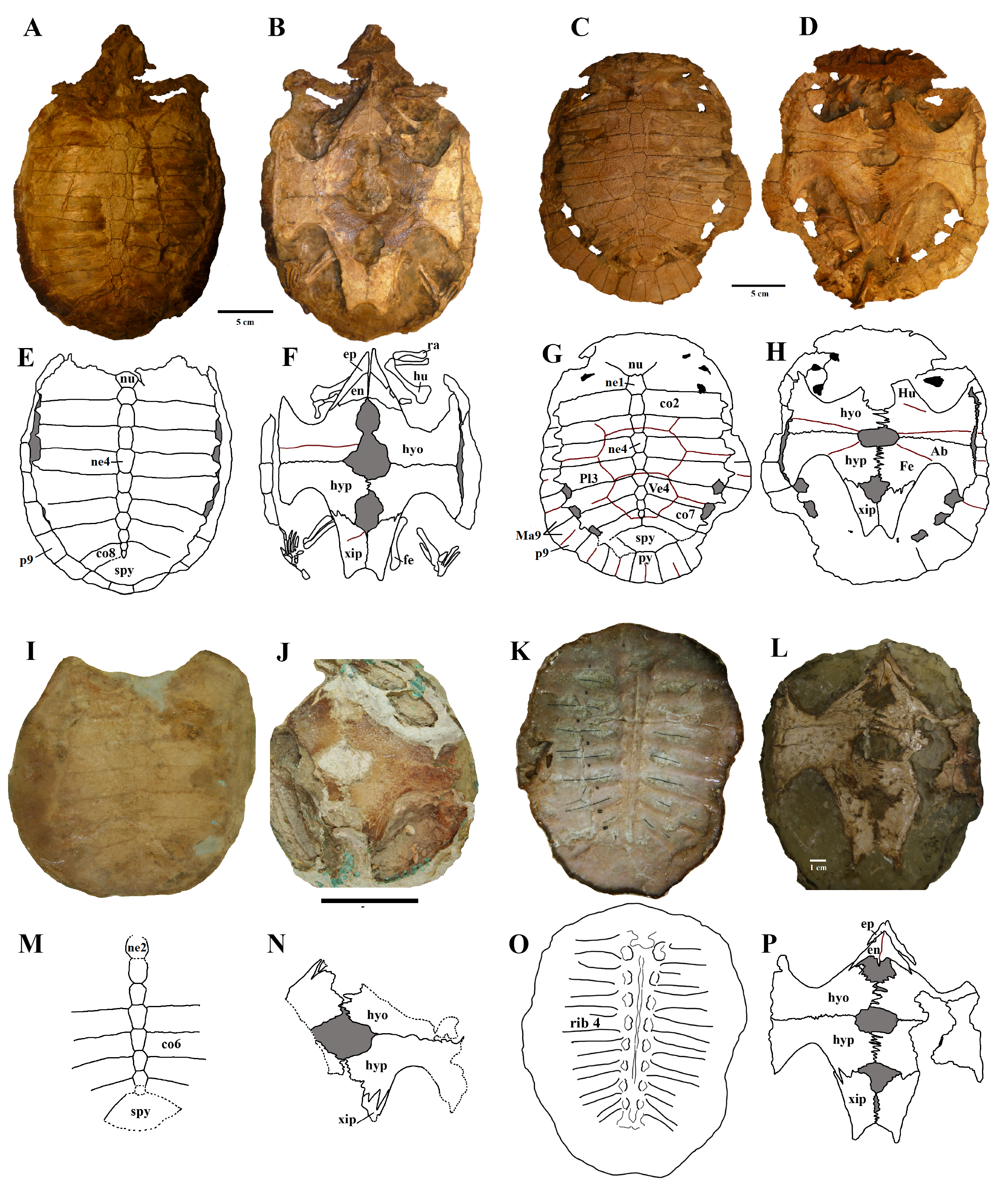

Supplement: Supplemental Information 5 — BSP 1977 I 1, A) carapace and B) plastron. Photo by Rodrigo V. Pêgas. BSP 1981 I 38, C) carapace and D) plastron. Photo by Rodrigo V. Pêgas. E), F), G), H), respective schematic drawings. MN 6637-V. I) carapace, encased in resin and J) plastron. Photo by Rodrigo V. Pêgas and Thales Nascimento. MN 6743-V. K) Impression of the carapace in internal view and L) plastron in visceral view. Photo by Rodrigo V. Pêgas and Thales Nascimento. M), N), O) and P), respective schematic drawings. Abbreviations: Ab – abdominal scute, co – costal, en – entoplastron, ep – epiplastron, Fe – femoral scute, fe – femur, Hu – humeral scute, hu – humerus, hyo – hyoplastron, hypo – hypoplastron, n – neural, nu – nuchal, pe – peripheral, py – pygal, ra – radius, rb – rib, spy – suprapygal, Ve – vertebral scute, xip – xhiphiplastron. [file peerj-08-9840-s005.png]

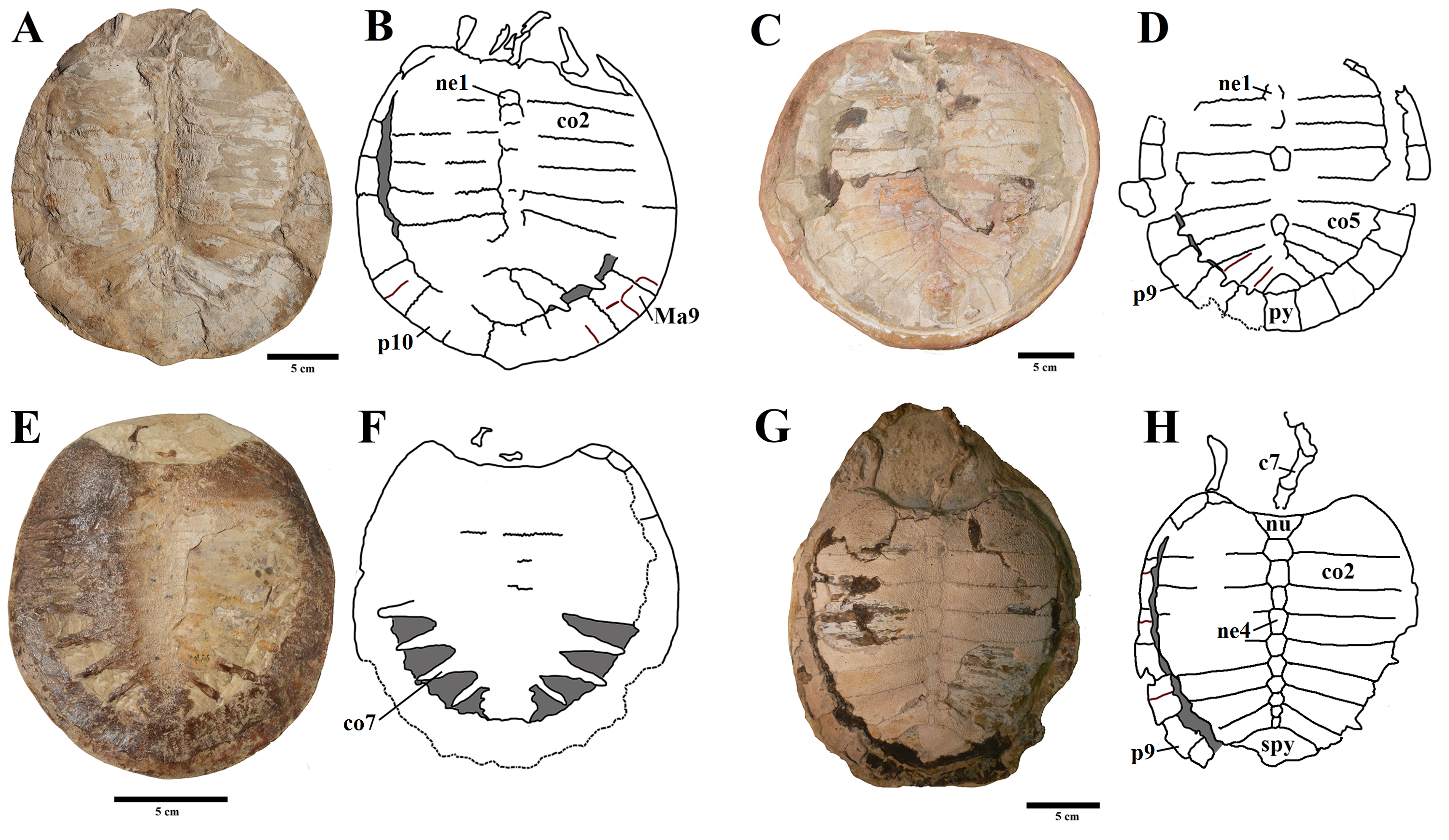

Supplement: Supplemental Information 6 — A) and B), DGM 1449-R. Photo by Rodrigo V. Pêgas. C) and D) MPSC R 134. Photo by Renan Bantim. E) and F) MPSC R 137. Photos by Renan Bantim. G) and H) SMNK no number. Photo by Rodrigo V. Pêgas. Abbreviations: co – costal, Ma – marginal scute, n – neural, nu – nuchal, pe – peripheral, py – pygal, spy – suprapygal. [file peerj-08-9840-s006.png]

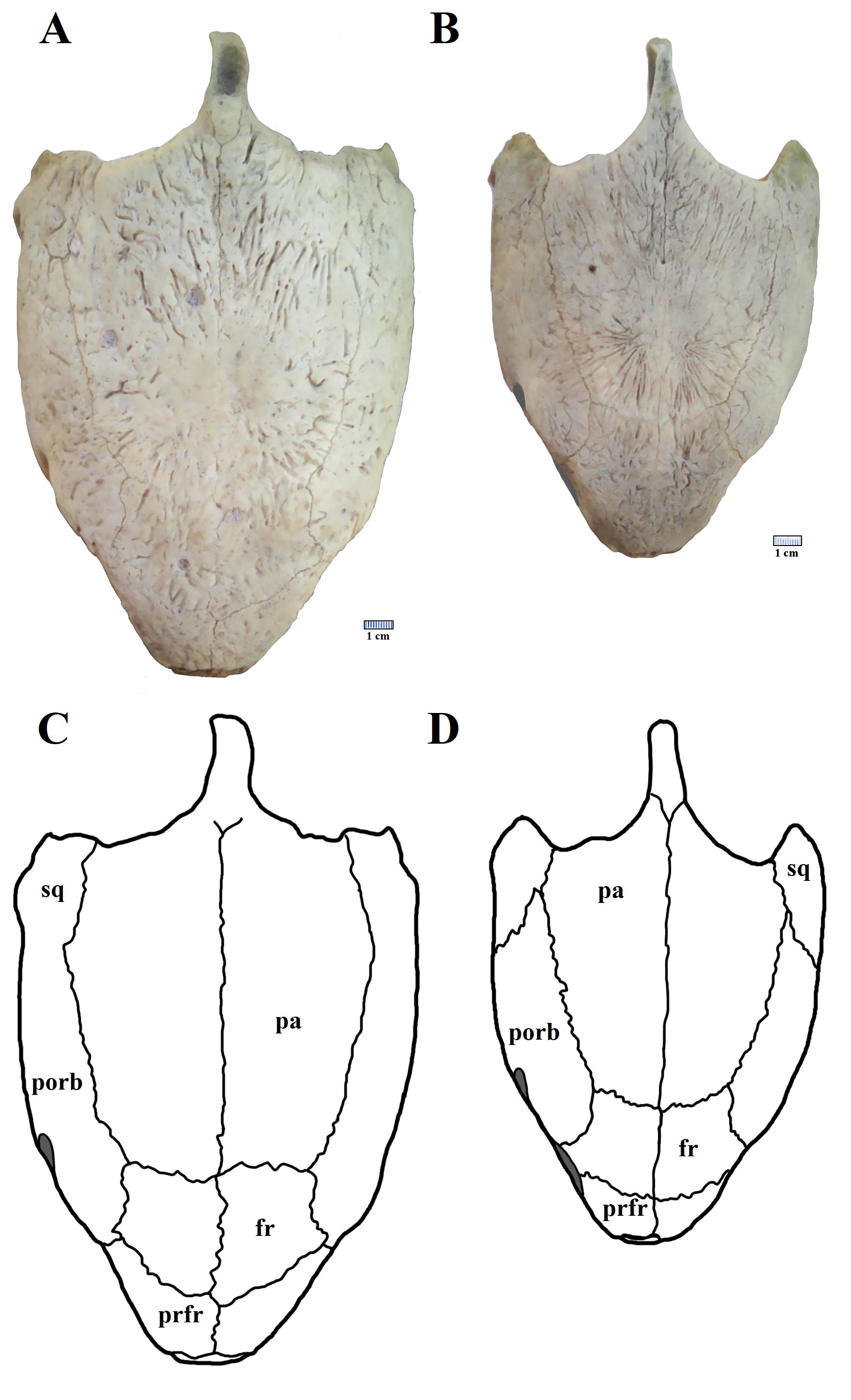

Supplement: Supplemental Information 7 — A), male, presenting contact between prefrontals and postorbitals. B), female with frontals separating prefrontals from the postorbitals and reaching the orbit margin. [file peerj-08-9840-s007.png]

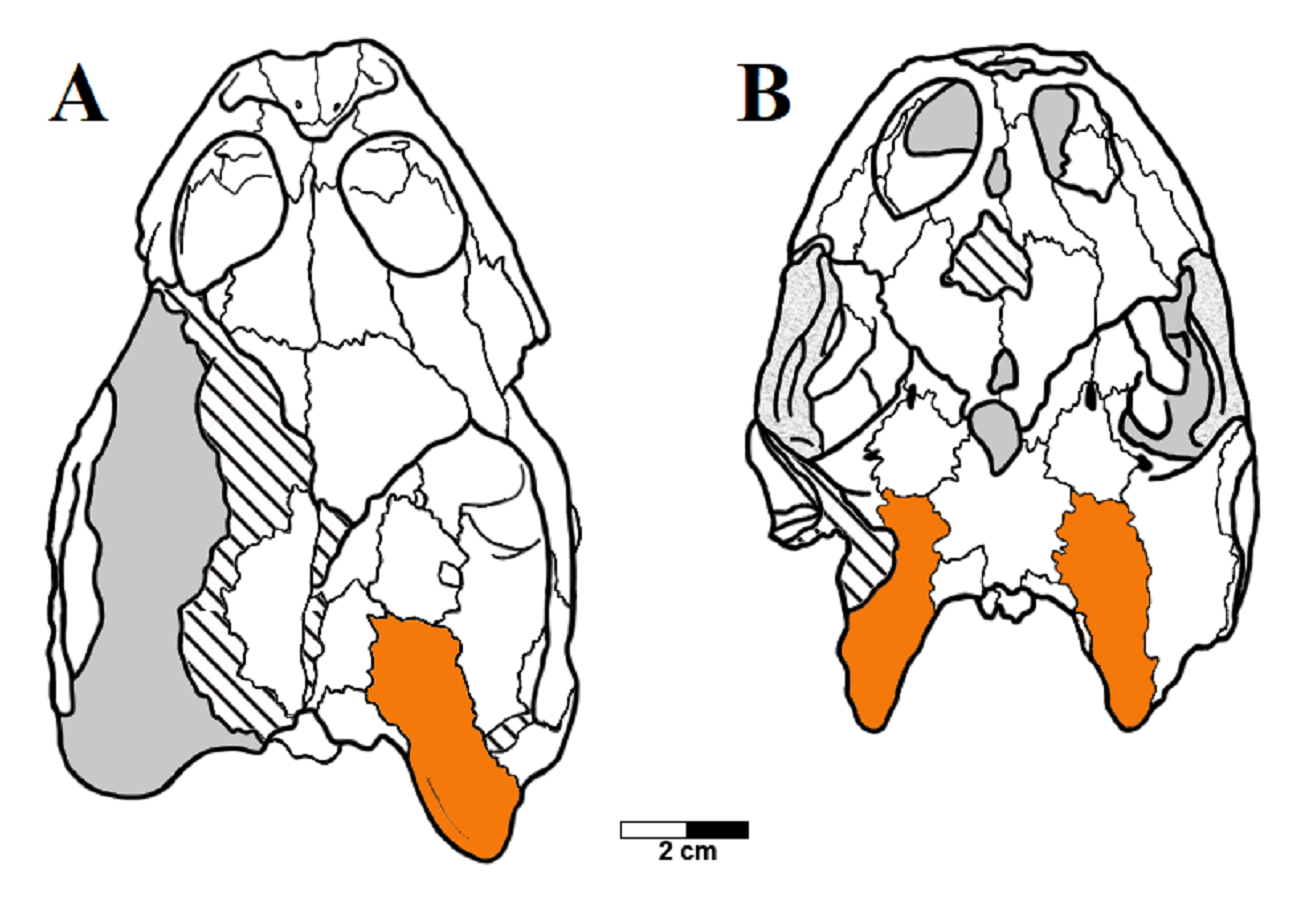

Supplement: Supplemental Information 8 — A) convex in THUg 1357 and B) concave in AMNH 24453. [file peerj-08-9840-s008.png]

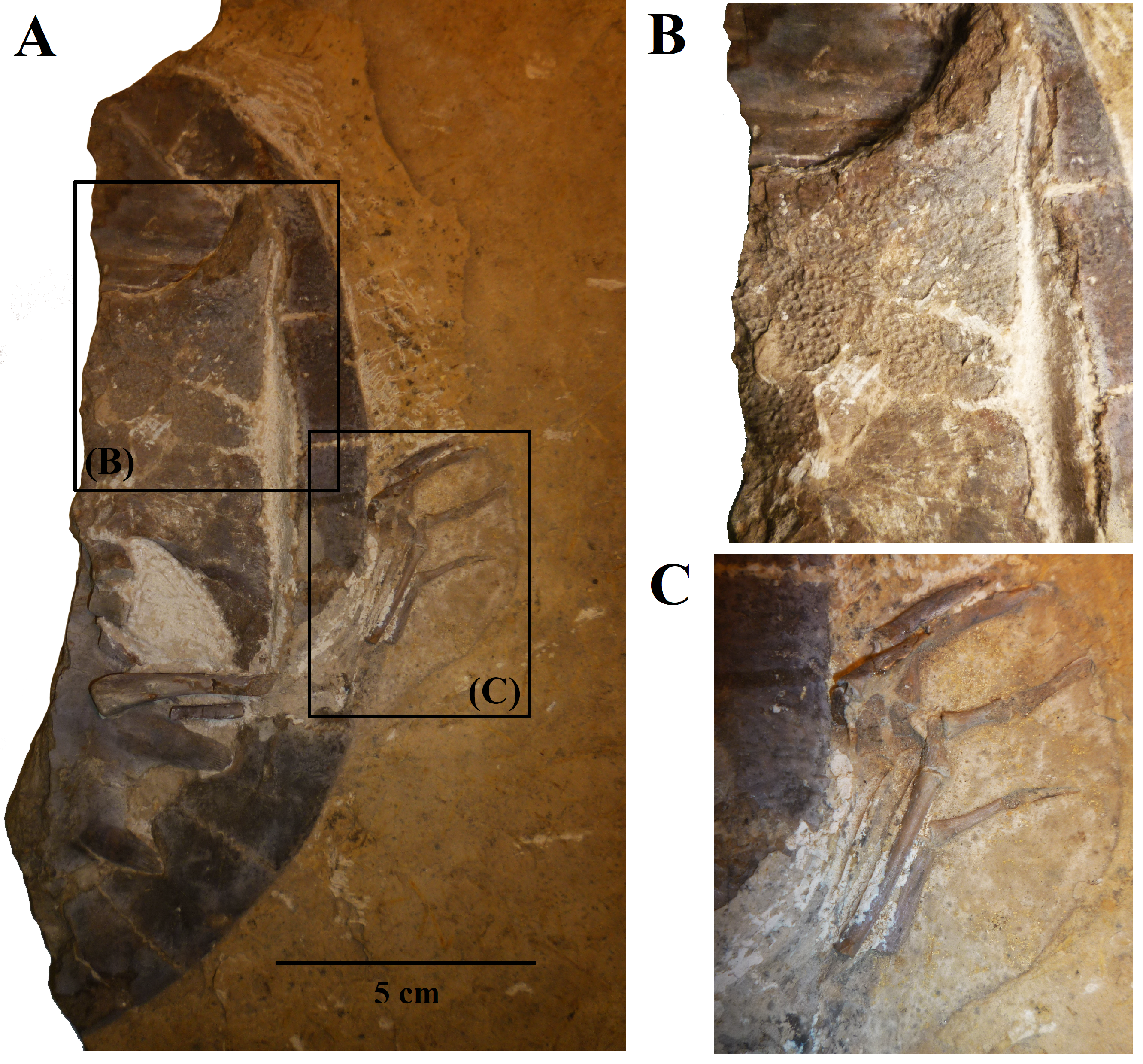

Supplement: Supplemental Information 9 — A) Whole specimen, ventral view. B) Plastral elements showing pitted ornamentation on the preserved ventral surface. C) Pes showing simple unguals. Photos by Rodrigo V. Pêgas. [file peerj-08-9840-s009.png]
